# Predicting human and viral protein variants affecting COVID-19 susceptibility and repurposing therapeutics

**DOI:** 10.1038/s41598-024-61541-1

**Published:** 2024-06-20

**Authors:** Vaishali P. Waman, Paul Ashford, Su Datt Lam, Neeladri Sen, Mahnaz Abbasian, Laurel Woodridge, Yonathan Goldtzvik, Nicola Bordin, Jiaxin Wu, Ian Sillitoe, Christine A. Orengo

**Affiliations:** 1 Institute of Structural and Molecular Biology, University College London, London, WC1E 6BT UK; 2 Department of Applied Physics, Faculty of Science and Technology, Universiti Kebangsaan Malaysia, Bangi, Malaysia

**Keywords:** COVID-19, Human genetic variation, SARS-CoV-2: human protein interaction, Protein structure complex, Functional family, CATH database, Protein binding affinity prediction, Immunity, Computational biology and bioinformatics, Protein structure predictions

## Abstract

The COVID-19 disease is an ongoing global health concern. Although vaccination provides some protection, people are still susceptible to re-infection. Ostensibly, certain populations or clinical groups may be more vulnerable. Factors causing these differences are unclear and whilst socioeconomic and cultural differences are likely to be important, human genetic factors could influence susceptibility. Experimental studies indicate SARS-CoV-2 uses innate immune suppression as a strategy to speed-up entry and replication into the host cell. Therefore, it is necessary to understand the impact of variants in immunity-associated human proteins on susceptibility to COVID-19. In this work, we analysed missense coding variants in several SARS-CoV-2 proteins and their human protein interactors that could enhance binding affinity to SARS-CoV-2. We curated a dataset of 19 SARS-CoV-2: human protein 3D-complexes, from the experimentally determined structures in the Protein Data Bank and models built using AlphaFold2-multimer, and analysed the impact of missense variants occurring in the protein–protein interface region. We analysed 468 missense variants from human proteins and 212 variants from SARS-CoV-2 proteins and computationally predicted their impacts on binding affinities for the human viral protein complexes. We predicted a total of 26 affinity-enhancing variants from 13 human proteins implicated in increased binding affinity to SARS-CoV-2. These include key-immunity associated genes (TOMM70, ISG15, IFIH1, IFIT2, RPS3, PALS1, NUP98, AXL, ARF6, TRIMM, TRIM25) as well as important spike receptors (KREMEN1, AXL and ACE2). We report both common (e.g., Y13N in IFIH1) and rare variants in these proteins and discuss their likely structural and functional impact, using information on known and predicted functional sites. Potential mechanisms associated with immune suppression implicated by these variants are discussed. Occurrence of certain predicted affinity-enhancing variants should be monitored as they could lead to increased susceptibility and reduced immune response to SARS-CoV-2 infection in individuals/populations carrying them. Our analyses aid in understanding the potential impact of genetic variation in immunity-associated proteins on COVID-19 susceptibility and help guide drug-repurposing strategies.

## Introduction

The COVID-19 pandemic has caused a major global health and socioeconomic burden since 2020. Many countries are still experiencing an intermittent rise in the number of infections due to the emergence of new Variants of Concern (VOCs) of SARS-CoV-2 and their sub-variants^1^. Although vaccines are now available, re-infection is common^2^. Various factors including ethnicity, age and clinical conditions have been proposed to be associated with an increased risk of infection^3–10^. In addition, increasing reports indicate that human genetic variation is a contributing factor for increased susceptibility and disease severity^11–13^.

Potential drug targets include human host proteins which interact with SARS-CoV-2^14,15^. In 2020, Krogan group identified a total of 332 human proteins that interact with SARS-CoV-2 proteins, using affinity purification followed by mass spectrometry (AP-MS)^15^. Subsequently, the presence of additional human proteins interactors of SARS-CoV-2 was also revealed by other studies based on techniques such as yeast two-hybrid assay, anti-tag coimmunoprecipitation, tandem affinity purification, pull down, structure-based studies (X-ray, NMR), etc.^14,16–19^ and are made available via dedicated protein-interaction resources such as IntAct (https://www.ebi.ac.uk/intact/home)^20^ and BIOGRID^21^. These studies indicate that interactor proteins in humans participate in a wide range of biological processes/pathways, including innate and adaptive immune pathways, lipid metabolism, cell adhesion, mRNA processing, among others^14,15,19,22^.

Innate immune suppression is known as one of the key characteristics of infections by SARS-CoV-2 and its VOCs. SARS-CoV-2 VOCs (namely Alpha, Beta, Gamma, Delta, and Omicron) are reported to exhibit increased interferon resistance as compared to the wild-type suggesting evasion of innate immunity is a driving force for SARS-CoV-2 evolution^23,24^. Furthermore, inborn variation in immunity-associated genes is reported to trigger susceptibility to COVID-19^25,26^. For example, rare variants in the Toll-like receptor 7 gene have been associated with increased severity and susceptibility of the COVID-19^12^. Likewise, various studies including those by the GeNOMICC-ISARIC consortium suggests association of both common and rare variants with increased severity of COVID-19^27–32^.

Though spike-ACE2 binding is the key entry mechanism used by SARS-CoV-2 for cell entry and infection, recent experimental studies indicate that SARS-CoV-2 also uses innate immune suppression as a strategy to speed-up entry and replication in the host cell^33,34^. Interactions such as SARS-CoV-2:ORF9b-human:TOMM70 and SARS-CoV-2:NSP1-human:NUP98 have been associated with innate immune evasion^14,34,35^. Further experimental studies have suggested involvement of other human protein interactors of SARS-CoV-2 associated with the immunity-associated pathways (e.g., IFIH1, ISG15, IFIT2)^33,34,36^.

In the case of SARS-CoV-2, spike-ACE2 is the most studied protein complex, where the impact of emerging variants in spike as well as natural human population variants in ACE2 have been computationally predicted by the Barton group and others^37–40^. Some of these predicted variants in ACE2 and spike proteins, have also been validated experimentally^37,40^. In this study, we focus on the impact of variants in immune-associated proteins and novel spike receptors (such as AXL and Kremen1) in humans on their binding to SARS-CoV-2 proteins. Such analyses could explain novel mechanisms by which SARS-CoV-2 proteins interfere with natural immune pathways and disrupt the system in humans.

Computationally predicted (docking-based) complexes for a subset of interactions identified from the Krogan study, are made available via several resources^41,42^. The Beltrao group has designed a resource called Mutfunc, which has predicted impacts of all possible single-amino acid substitutions in SARS-CoV-2 proteins, using known 3D structure data (http://sars.mutfunc.com/home^43^). Similarly, the Ascher group developed the COVID-3D resource, to analyse > 11,000 SARS-CoV-2 variants and predicted the impact of missense variants using spike-ACE2 complex^39^. More recently, a powerful AlphaFold2-based protein structure prediction method has been developed for modelling protein–protein complexes which facilitates modelling of interactions between SARS-CoV-2 and human proteins which have yet to be experimentally characterized, and with improved accuracy compared to other approaches^44–48^.

In this study, we analysed the impact of missense coding variants in human and viral proteins occurring at protein–protein interfaces, using a curated dataset of 19 immune-associated SARS-CoV-2:human protein 3D-complexes, obtained from the Protein Data Bank^49^ and models built using AlphaFold2-multimer^44,46^. For the human proteins, we obtained population variants from various databases including gnomAD (^50^ https://gnomad.broadinstitute.org) and GenomeAsia 100K^51^. For the viral proteins, we obtained mutation data from ViralZone (https://viralzone.expasy.org/), CoV-Glue (https://cov-glue.cvr.gla.ac.uk/) and COG-UK (https://sars2.cvr.gla.ac.uk/cog-uk/)^52–54^. The impact of variants on binding affinity of the complexes was computationally predicted using a state-of-the-art program (mCSM-PPI2)^55^. The structural and functional impact of the predicted affinity-enhancing variants was analysed in the context of proximity to known functional sites such as protein–protein interfaces, ligand or substrate -binding sites and predicted sites identified using conserved positions in Functional Families in the CATH database (i.e., CATH-FunFams)^56–58^. CATH-FunFams represent functionally coherent groups i.e., members of a CATH-FunFam have high structural and functional similarity^56^. Finally, the human proteins implicated in enhanced SARS-CoV-2 binding are mapped onto protein networks to understand biological pathways/processes associated with the network modules. We then studied whether these proteins are associated with CATH-FunFams that are enriched in small molecules or drugs from ChEMBL^59,60^.

In summary, we analysed the impact of missense coding variants occurring at protein–protein interfaces in a total of nineteen 3D complexes of human proteins and SARS-CoV-2 interactors. A total of 26 affinity-enhancing variants from 13 human proteins (namely TOMM70, IFIH1, IFIT2, ISG15, RPS3, PALS1, NUP98, AXL, ARF6, KREMEN1, TRIMM, TRIM25 and ACE2) were predicted to enhance binding affinity to their interacting proteins in SARS-CoV-2. Our study sheds light on affinity-enhancing variants in immunity-associated proteins; their frequencies in gnomAD populations; their impact on protein structure and function and the populations more likely to be susceptible to COVID-19 infection. We provide computational evidence that the predicted affinity-enhancing variants in human proteins could promote binding to SARS-CoV-2 proteins, instead of their natural protein partners or substrates in immune pathways, thereby hampering the normal antiviral activity and leading to increased susceptibility. Protein Functional families associated with three proteins (IFIH1, AXL and ARF6) are associated with small molecule inhibitors and their potential applications in drug-repurposing in discussed.

## Materials and methods

### Compilation of interactors associated with SARS-CoV-2 immunity

The dataset of human proteins interacting with SARS-CoV-2 proteins, was compiled using the COVID-19 UniProtKB resource (^61^; https://covid-19.uniprot.org/uniprotkb?query=*) and IntAct database (^20^; https://www.ebi.ac.uk/intact/home). IntAct provides a COVID-19 dataset of SARS-CoV-2: human protein interactors which is based on interactions reported from experimental studies. For every protein–protein interaction, IntAct assigns an MIscore (ranges from 0 to 1) based on (i) the type of the experimental detection method (ii), the number of associated publications and (iii) the interaction types (such as direct association, physical association). The IntAct recommended MIscore threshold of 0.45 was used to exclude low-confidence interactions^62^.

Thus, the dataset of a total of 536 high-confidence (MI-score ≥ 0.45) interactions are considered for subsequent analyses. We further filtered immunity-associated human proteins by mapping specific GO terms associated with immunity using UniProt (i.e., GO:0002250 GO:0002218, GO:0002376, GO:0045087, GO:0045089, GO:0060337, GO:0050776 and GO:0006955)^61,63^; and by mapping the UniProt IDs to InnateDB database (http://innatedb.sahmri.com/^64^. We also curated available literature-based evidence specifying a COVID-19-associated immunological role associated with interacting pairs of proteins in dataset.

### Functional families in the CATH database and conserved functional sites

The CATH database provides a hierarchical structural classification of protein domains into Class (C), Architecture (A), Topology (T) and Homologous Superfamily (H). In CATH, protein domains are classified into superfamilies where there is strong evidence of an evolutionary relationship via structure and sequence similarity^65,66^. Within each superfamily, sequences are sub-classified using an agglomerative clustering protocol followed by an entropy-based segregation of functionally coherent subgroups known as Functional Families (CATH-FunFams)^56^. The conserved sites obtained from CATH-FunFams have been shown to be enriched in known protein functional sites^56,57^.

For the dataset of shortlisted human proteins and their SARS-CoV-2 interactors, we identified CATH-FunFams, and subsequently conserved sites as follows:

#### Identification of CATH-FunFams

We scanned the sequences of human and their interacting SARS-CoV-2 proteins against HMMs of FunFams in CATH v4.3 (https://www.cathdb.info/), using HMMsearch (E-value < 1e^−3^)^67^. We then processed the output of HMMsearch using cath-resolve-hits, an in-house tool built to obtain the best non-overlapping set of domain matches^67,68^.

#### Identification of conserved sites using CATH-FunFams

Using the matching CATH-FunFams, we identified conserved sites using Scorecons, an entropy-based method^69^. The multiple sequence alignment (MSA) program, namely MAFFT is used to construct an MSA from seed sequences within a FunFam^68^. The Scorecons program is then applied to each MSA to determine an overall measure of sequence diversity called Diversity of Positions score (DOPs). DOPs captures the amount of diversity in an MSA by considering all the different conservation scores, and their frequencies, and provides a value from 0 (i.e., zero diversity) and 100 (i.e., high diversity). Only MSAs with a DOPs score ≥ 70 were considered for further analyses. The Scorecons program also provides the degree of conservation of each position in the MSA. Thus, for each column in a CATH-FunFam based MSA, the Scorecons program provides a conservation score ranging from 0 (i.e., not conserved) to 1 (i.e., completely conserved). The sites belonging to alignment positions with Scorecons-based score ≥ 0.90 are used for analyses and are referred to as Scorecons90 in this manuscript.

In addition to Scorecons method by Valdar, we used other conservation scoring methods such as Shenkin^70^ and Thompson^71^ (available from Jalview^72^ (https://www.jalview.org/) for detecting conservation scores. We used the consensus among the two methods for predicting conserved sites (score ≥ 0.90).

### Compilation of missense coding variants in human and SARS-CoV-2 proteins

#### Human protein variants

For the human genes, missense coding variants from canonical transcripts were obtained from the Genome Aggregation Database (gnomAD, v2.1.1; the recommended version for coding region analyses) (https://gnomad.broadinstitute.org/)^50^. We compiled ancestry (i.e., ethnic population) information available from gnomAD, using VarSite^73^. GnomAD provides ancestry for the following populations: African/African American (afr), American Admixed/Latino (amr), Amish (ami), Ashkenazi Jewish (asj), East Asian (eas), South Asian (sas), Finnish (fin) and Non-Finnish European (nfe). If individuals did not unambiguously cluster with any of these populations in a principal component analysis (PCA), gnomAD classifies them as "other" (oth). GnomAD v2 also provides sub-continental information for the East Asian cohort (Japanese, Koreans) and Non-Finnish European (Bulgarian, Estonian, Swedish, North-Western European, Southern European) populations.

#### SARS-CoV-2 protein variants

For each of the associated interactor proteins in SARS-CoV-2, a non-redundant set of mutations in strains of SARS-CoV-2 (including VOCs and Variants of Interest) was compiled from resources such as ViralZone^54^, COG-UK (^52^ https://sars2.cvr.gla.ac.uk/cog-uk/), and CoV-Glue (https://cov-glue-viz.cvr.gla.ac.uk/)^53^.

### Functional site data from CATH-FunVar (Functional Variation) protocol

All gnomAD missense variants in the interactor human genes were processed using a modified version of the in-house FunVar protocol to identify variants occurring near known and predicted functional sites. Predicted sites comprise CATH-FunFam-based conserved residues, i.e., highly conserved residues identified by the Scorecons program (i.e., Scorecons90 sites, as described above).

Known functional sites were compiled from existing resources and include: known ligand and nucleic acid binding sites from BioLip^74^; protein–protein interface (PPI) residues from PDBSum^75^; catalytic sites from M-CSA and VarMap^76,77^ and annotated functional sites in UniProt resource.

Spatial proximity of each gnomAD variant to these sites was found by mapping each variant to a representative domain from the corresponding FunFam in CATH v4.3. Functional sites were similarly mapped to FunFam representative domains, allowing detection of variants occurring on or near (within 5 Å) of any of the functional sites.

For each variant, the Grantham score (https://gist.github.com/danielecook/501f03650bca6a3db31ff3af2d413d2a) was calculated to identify variants having a significant change in physico-chemical properties (such as volume, polarity), as compared to that of wild-type residues.

Finally, each variant was assigned a simple functional impact score (from 1 to 5) by counting each of the impacts—scoring 1 for each of: high Grantham score; variant is a catalytic site; variant lies on a known functional site; variant near (5 Å) a known site; variant is on a conserved predicted (Scorecons90) site. Additionally, CATH-FunVar reports impact scores from CADD^78^ and SIFT^79^.

### Three-dimensional (3D) structures of complexes

3D structures of complexes are available for 10 interactions as follows—human:TOMM70-SARS-CoV-2:ORF9b [PDB ID: 7KDT], human:ISG15-SARS-CoV-2:PLpro [7RBS], human:RPS2-SARS-CoV-2:NSP1 [6ZMT], hRPS3-SARS-CoV-2:NSP1 [6ZMT], human:Ubiquitin-SARS-CoV-2:Plpro [7RBR], human:APOA1-SARS-CoV-2:ORF3a, human:PALS1-SARS-CoV-2:E, human:NUP98-SARS-CoV-2:ORF6 [7VPH] and humanRAE1-SARS-CoV-2:ORF6 [7VPH] and human:ACE2-SARS-CoV-2:spike [wild-type (6M0J, 7A95); Alpha (7EDJ), Beta (7V7Z), Gamma (7V83), Delta (7V89), Omicron (7T9K), BA.1 (7XO6), BA.2 (7XB0, 7XO8), BA.3 (7XB1)].

For the remaining interactions with no available structures of complexes in the PDB, we predicted models using AlphaFold2-ptm and AlphaFold2-multimer(v1)^44,46^, as described below.

#### Modelling complexes using AlphaFold2-ptm and AlphaFold2-multimer

Prior to modelling protein–protein complexes, we excluded some interactions from the modelling procedure due to the following reasons—we excluded proteins of very short lengths such as ORF3b (22aa residues long) and proteins for which high-quality models are not built by AlphaFold2 (https://alphafold.ebi.ac.uk/).

We modelled the remaining complexes using the AlphaFold2-ptm and alphafold-multimer(v1) protocols, which were made available in March, 2022 (^44,46,80^; https://github.com/sokrypton/ColabFold). We built models using both the AlphaFold2_ptm and AlphaFold2-multimer(v1) methods and then selected a model from one of these methods, whichever had the best interface quality (see “Results”, Table 1).

**Table 1:** Summary of human: SARS-CoV-2 protein complexes used for the study.

SARS-CoV-2 protein	Human protein interactor	Source of Complex	Interface pLDDT score	Interface PAE score	PIZSA Interface stability Z-score (≥ 1.5 = stable)	PRODIGY ΔG (kcal/mol)
ORF9b	TOMM70	PDB [ID:7KDT]	n/a	n/a	2.507	− 17.4
NSP1	RPS2	PDB [ID: 6ZMT]	n/a	n/a	2.283	− 4.6
NSP1	RPS3	PDB [ID: 6ZMT]	n/a	n/a	2.805	− 4.5
Spike-RBD	ACE2	PDB [ID:6M0J]	n/a	n/a	2.582	− 11.9
ORF6	NUP98	PDB [ID: 7VPH]	n/a	n/a	2.780	− 6.1
ORF6	RAE1	PDB [ID: 7VPH]	n/a	n/a	2.161	− 8.8
ORF3a	APOA1	PDB [ID: 7KJR]	n/a	n/a	2.569	− 15.6
E	PASL1	PDB[ID:7M4R]	n/a	n/a	1.685	− 6.6
PLPro	ISG15	PDB[ID:7RBS]	n/a	n/a	2.046	− 13.4
PLPro	UBB	PDB[ID:7RBR]	n/a	n/a	2.424	− 10.4
PLPro	IFIT2	AlphaFold2-ptm	72.82	9.86	2.460	− 11.3
PLPro	IFIH1	AlphaFold2-multimer(v1)	83.00	10.00	2.375	− 10.1
N	TRIM25	AlphaFold2-ptm	93.15	2.87	2.24	− 9.1
NSP7	SCRB1	AlphaFold2-multimer(v1)	83.87	4.27	2.144	− 8.0
Spike-RBD	KREMEN1	AlphaFold2-ptm	81.02	4.74	2.148	− 10.2
NSP15	ARF6	AlphaFold2-ptm	82.61	7.20	2.063	− 7.5
NSP14	TRIMM	AlphaFold2-ptm	81.24	7.58	1.974	− 8.8
Spike-NTD	AXL	AlphaFold2-ptm	75.31	8.55	2.321	− 10.6
ORF7b	UN93B	AlphaFold2-ptm	72.77	9.45	2.488	− 5.1

The table lists 3D complexes used in this study. We outline the source, quality metrics such as Interface pLDDT (≥ 70), Interface PAE (≤ 10), PIZSA Interface stability Z-score (≥ 1.5 indicates stable interface) and PRODIGY binding energy (ΔG ≤ − 4.3 kcal/Mol, cutoff chosen based on experimental binding energy values reported in the PRODIGY^82^. The literature supporting biochemical evidence of protein interaction is also cited in Supplementary file 2. For modelled complexes, we first built models using the AlphaFold2 (ptm method) and AlphaFold2-multimer(v1) methods and then selected the model from one of these methods which had the best interface quality.

High-confidence models were chosen where models have overall pLDDT (predicted local difference distance test) ≥ 70 as well as pTM-Score (predicted TM-score) ≥ 70^44,46^. We further filtered complexes on the basis of the interface quality i.e., interface-pLDDT (i.e., those with < 70 were excluded) and interface-PAE (predicted alignment error > 10 were excluded) and by manually inspecting the domain interface regions (i.e., we excluded models where we observed erroneous overlapping/entangled interface). We performed additional quality checks such as verifying the interface stability score using the PIZSA method (which calculates protein interaction Z-Score; ≥ 1.5 indicates stable interface)^81^ and predicted binding affinity of the complexes by the PRODIGY (PROtein binDIng enerGY prediction) method^82^. Using the 3D-structure of the protein–protein complex as an input, the PRODIGY server (https://wenmr.science.uu.nl/prodigy/) predicts the binding affinity of the complex and provides the predicted value of the binding free energy (ΔG) in kcal/mol. PRODIGY is benchmarked using an experimental dataset comprising 144 non-redundant protein–protein complexes with known 3D structures (of both bound and unbound). The resultant high-confidence models predicted in this study along with their quality metrics are given in “Results” section, Table 1.

### Extraction of interface residues using complexes

After the selection of the complexes, we extracted interface residues as follows:

#### Directly-contacting (DC) residues

For the PDB complexes, we extracted directly-contacting (DC) interface residues made available by PDBSum^75^. PDBSum calculates the protein–protein interfaces using the NACCESS program (http://www.bioinf.manchester.ac.uk/naccess/). For the modelled complexes, we extracted interface residues by selecting residues from interacting chains with heavy atom distances ≤ 4 Å)^83,84^. Human protein missense variants in gnomAD that occur in DC interface residues are referred to as ‘DC-variant’ residues.

#### Secondary shell residues

Residues that occur within 5 Å from the DC interface are considered to be residues in the secondary shell and variants at these residue positions are likely to influence binding^38,85^ and are referred to as ‘DCSS-variants’.

### Predicting the impact of variants in human and viral proteins on the binding affinity of the complexes

For the filtered dataset of complexes (experimental and predicted), we applied the mCSM-PPI2 program^55^ to identify human and viral missense variants which could significantly impact binding-affinity of interacting proteins. We analysed the impact of missense variants reported in gnomAD that occur at directly contacting interface (DC-variant) and secondary shell (DCSS-variant) residues.

The mCSM-PPI2 program, developed by the Ascher lab, was shown to be the top-performing method when compared to 26 other methods in CAPRI (round 26) blind tests^55^. mCSM-PPI2 is based on a graph-based structural signature framework with evolutionary information, inter-residue non-covalent interaction networks analysis plus computed energetic terms, providing an optimised overall prediction performance. It was used to predict change in binding affinity (i.e., mCSM-PPI2 ΔΔG^Affinity^ in kcal/mol) for each mutation.

A positive mCSM-PPI2 ΔΔG^Affinity^ score (> 0 kcal/mol) indicates that the mutation is stabilising the interaction whereas a negative ΔΔG^Affinity^ score (< 0 kcal/mol) indicates a destabilising effect^55^. Where available, we compiled evidence from experimental studies reporting experimental mutagenesis and binding affinity kinetic assays^14,37^ to choose our ΔΔG thresholds. For example, in the case of spike-ACE2 complex, the mutations K26R in ACE2 and S477N in the spike protein are reported to increase the binding affinity of the spike-ACE2 complex using kinetic assays^37,38^, and these are predicted to have mCSM-PPI2 ΔΔG^Affinity^ of 0.12 kcal/mol and 0.5 kcal/mol respectively. Likewise, experimental alanine scanning mutagenesis studies in the ORF9b-TOMM70 complex indicates that a E477A mutation in TOMM70 and a S53A mutation in ORF9b significantly reduced the binding affinity of the interaction^14^, and these are predicted to have a mCSM-PPI2 ΔΔG^Affinity^ of < − 0.5 kcal/mol. Therefore, we used the confidence cut-offs of ΔΔG^Affinity^ ≤ − 0.5 kcal/mol (for destabilising; affinity-reducing) and ≥ 0.5 kcal/mol (for stabilising; affinity-enhancing), to analyse the structural impact of gnomAD variants in this study, while we also provide a catalogue of other mutations predicted to have scores ranging from 0 to 0.490 kcal/mol (see “Results” section). Through in silico saturation mutagenesis, we further confirmed that the mCSM-PPI2 method does not show bias towards predicting positive ΔΔG^Affinity^ scores and corresponds well to observed changes in amino acid properties for mutated residues.

To evaluate the reliability (reproducibility) of the binding affinity values predicted by mCSM-PPI2, we computed 95% confidence interval for affinity-enhancing variants (ΔΔG^Affinity^ ≥ 0.5 kcal/mol). We generated 10 different versions for each of the SARS-CoV-2:Human protein complex structure, using GalaxyrefineComplex software^86^, which is specifically designed for structure refinement of protein–protein complexes. Its refinement protocol involves both an initial local energy minimization and a 1.2-ps molecular dynamic (MD) relaxation with a 4-fs time step. Thus, incorporation of MD relaxation step allows some physical space for atomic fluctuations, facilitating further conformational sampling. Particularly, relaxation of the input complex structure is driven by side-chain repacking of interfacial residues, which ensures overall conformational changes. Side-chain repacking and MD relaxation is repeated 22 times (please refer to Heo et al.^86^, for details).

#### Analysis of predicted affinity-enhancing variants

As the affinity-enhancing variants could be associated with increased risk of susceptibility/infection, we closely examined their 3D structural impact on molecular interactions and characterised these variants in the context of known and predicted functional sites (see FunVar section above) and using UniProt site annotations. Variants are mapped on 3D-complexes and visualized using UCSF Chimera^83^. We further analysed their associated population allele frequencies in gnomAD as well as other databases such as Indigenomes^87^, SweGen^88^, GenomeAsia 100K^51^, jMORP^89^ and the NIH-funded research hub called All of Us (^90^ https://databrowser.researchallofus.org/).

Finally, for affinity-enhancing variants we analysed pathogenicity scores by mutpred2 (score ≥ 0.50), CADD (score ≥ 20) and SIFT (deleteriousness score ranges from 0 to 0.05). MutPred2 is a machine learning-based method, which uses structural and functional features (e.g. secondary structure, allostery, binding site data, etc.) to provide pathogenicity score. SIFT relies on sequence conservation information and considers position and the type of amino acid change. CADD combines multiple sources of information (genomic features, biochemical activity, and scores from other predictors and applies a machine learning based scoring system. These methods were chosen as the study by Pejaver group showed that Mutpred2 performs better than other predictors while CADD and SIFT were the second and third best performing tools^91^. We used Dynamut2^92^, to analyse impact of affinity-enhancing variants on protein stability in human proteins (https://biosig.lab.uq.edu.au/dynamut2/).

### Network mapping and enrichment analysis

#### Identification of network modules

The human proteins containing affinity-enhancing variants with putative impact on SARS-CoV-2 binding, were mapped to ConsensusPathDB (CPDB) and STRING database (STRINGdb) protein–protein interaction networks^93,94^. Interactions from STRINGdb were filtered to include only those with confidence values ≥ 0.2 to optimize signal-to-noise ratio. A module detection algorithm (M1) was applied to the network using the MOdularising NEtwork Toolbox that adopts a multiresolution approach to combine optimization algorithms to improve modularity^95^.

#### Enrichment analyses

Pathway enrichment analysis was performed using g:profiler (https://biit.cs.ut.ee/gprofiler/gost^96^), a public webserver using Ensembl and Ensembl Genomes to identify significantly enriched terms in Gene Ontology (GO) biological processes, KEGG and Reactome databases^97–99^. Pathways were ranked by significance (p < 0.01) and the most significant term per database for the protein or each module was taken as the representative pathway. Where necessary, Ensembl identifiers with the most GO annotations were selected according to the default g:profiler function. Values and pathways for STRINGdb and CPDB modules were retained.

### Identification of druggable functional families in CATH database

The human proteins with affinity-enhancing variants, were mapped to the CATH functional families (CATH-FunFams) that are linked with small molecule information from ChEMBL^59,60^. For the associated human proteins, the druggability score of the protein–protein interface region was analysed using the CavityPlus and CASTp programs^100,101^. Where the protein–protein complex is significantly predicted to contain druggable cavity, we performed docking using AutoDockTools^102^.The ligand molecule was extracted from the PubChem database^103^. The predicted druggable cavity site by CavityPlus was used to dock the drug molecule.

**Figure 1:**
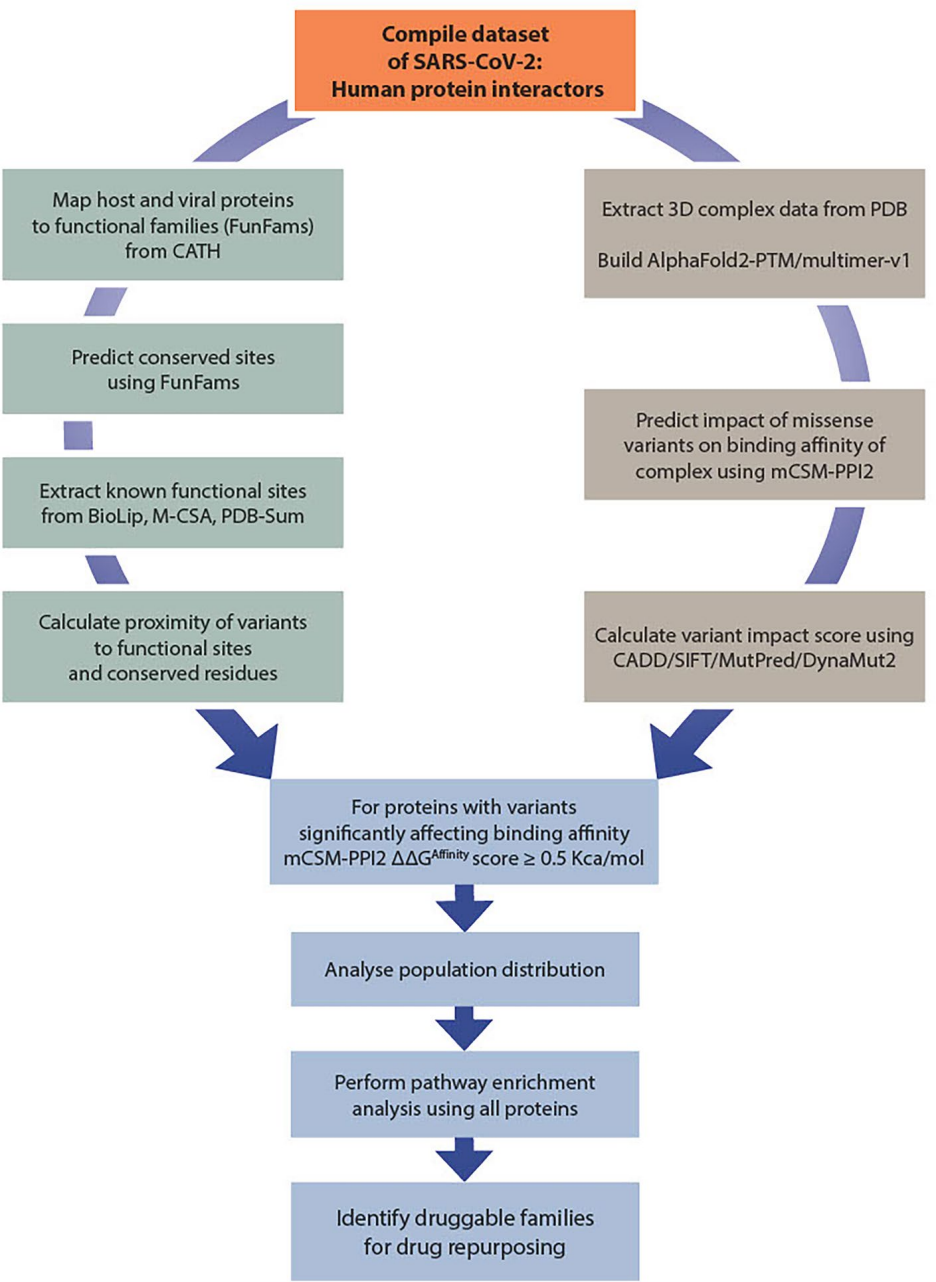
Flow-chart of the methodology used in this study. The dataset of SARS-CoV-2: human protein interactors is analysed using variants from gnomAD (in case of human proteins). The in-house CATH-FunVar (Functional Variation) pipeline was used to extract all annotations on known and predicted functional sites. For the missense coding variants from gnomAD, the impact of binding affinity of the complex was analysed using mCSM-PPI2 program. Affinity-enhancing variants were analysed using functional sites, and population data from gnomAD. The human proteins predicted to contain affinity-enhancing variants are used for pathway enrichment and identification of CATH functional families (CATH-FunFams) linked with small molecules/drugs from ChEMBL.

The methodology used in the study is summarised in Fig. 1.

## Results

### Dataset of SARS-CoV-2: human protein interactors, 3D-complexes and missense variants

As described in Methods, out of the 536 high-confidence (MIscore ≥ 0.45) protein–protein interactions, we curated 94 human proteins involved in SARS-CoV-2 infection and immunity by identifying those with immunity-associated GO terms. For the curated dataset of 94 human proteins, we compiled information from multiples sources: the interactor protein in SARS-CoV-2, IntAct MIscore^20^, UniProt accession IDs^61^, immune-associated GO terms, literature evidence linked to the interaction in IntAct, literature-based evidence for COVID-19 immune association, CRISPR-association (obtained from BIOGRID-ORCS; https://orcs.thebiogrid.org/^21^) and gene-expression (obtained from SARS-COVIDB; https://sarscovidb.org/^104^). This information is provided in the Supplementary files 1 and 2.

In total, the 94 human proteins were associated with 110 SARS-CoV-2:human interactions (Supplementary file 2). Experimental 3D structures for 10 interactions were available in the PDB, with a further 9 high-quality models predicted using AlphaFold2-multimer/ptm method, as summarised in Table 1. Thus, a total of 19 protein 3D structural complexes were used for subsequent analyses.

For the human proteins from this curated dataset of 19 complexes (Table 1), we analysed a total of 468 missense variants from gnomAD; that occur at directly contacting (DC) residues in the interface of the complexes (DC-variants, 131 in total) as well as those that occur within 5 Å from the DC residues i.e., secondary shell (DCSS-variants, 337 in total).

A total of 26 variants from 13 human proteins, are predicted to significantly enhance binding affinity to their SARS-CoV-2 protein partners by the mCSM-PPI2 program (ΔΔG^Affinity^ ≥ 0.5 kcal/mol), as given in Table 2.

**Table 2:** 13 human proteins with variants predicted to have significant impact on binding affinity to SARS-CoV-2 interactor proteins (by mCSM-PPI2 program^55^).

Name of the complex: human (SARS-CoV-2 protein)	Affinity-enhancing variants in human proteins (ΔΔG^Affinity^ ≥ 0.5 kcal/mol) (stabilizing mutations)	Affinity-reducing variants in human proteins (ΔΔG^Affinity^ ≤ − 0.5 kcal/mol) (de-stabilizing mutations)	Other affinity-enhancing variants in human proteins (0 < ΔΔG^Affinity^ < 0.49 kcal/mol)
hTOMM70 (ORF9B)	**V556L, K576R, A591T, V514I, A483T**	I412T, Q477G, H515Q, L584M, I 554T, D589N, L111P, F155L, G530S, D397N	A582V
hISG15 (PLPro)	**L121Q**	L60V, G128V, L28M, F122L, P130T, E127G, M23V, N151D, L85P, M23T, R155W,N151K, R155Q	S21N, Q55R, Q118R,V148L,A30V,D56Y, Q31H
hIFIH1 (PLpro)	**Y13N, S16L**	–	L120F, F107S, L121F, M95
hIFIT2 (PLpro)	**L373F, K221E, A319S, A319T, L373F, Y383F**	N300K, R376Q, R406S, L375V, R376P, Q384K, L323P, G398V, F380L	Q381H, R292K, P190Q, A391V
hRPS3 (NSP1)	**V164I, I99F**	R106C, L86H, Y34H, V153A	E81Q, A52T, I223V, A114P,
hNUP98-hRAE1(ORF6)	**NUP98: T190S**	–	NUP98: K185R, I162V RAE1: A275V, S311L
hARF6 (NSP15)	**L166F**	I29V, L33V, Y78C	N56H, D68H, I96F
hTRIM25 (N)	**A466T**	V470M, F615L	C475Y, P490L, I457V, A471S, K458E, C506S
hTRIMM (NSP14)	**I105F**	–	Q160R, H223Y, K146R, A144E, I13F
hAXL (spike-NTD)	**V38M**	L54Il, Q57K	A79E
hKremen1 (spike-RBD)	**Y66H, V189I**	Y98C, C200Y, C86Y, E187K, V93M, E187K, V93M, H196L, Y167C, Y107H, G166D, W108R	–
hACE2 (spike-RBD)	G326E*	G352V, D355N, L585P	A501T*, K26R*, S19P*, K26E*
hPALS1(E)	**L321F**	–	–

The first column lists the name of complexes in the following format—human protein (SARS-CoV-2 interactor protein). For every complex, the variants in human proteins that are predicted to enhance binding affinity with ΔΔG^Affinity^ score ≥ 0.5 kcal/mol are indicated in bold. The variants that are predicted to increase binding affinity but with lower scores ranging from 0 < ΔΔG < 0.49 kcal/mol are provided in the last column. *Indicates that the ACE2 variants reported in our study are also noted to enhance binding affinity in previous studies^38,40^.

To further validate the reproducibility of the binding affinity scores by the mCSM-PPI2, we calculated 95% confidence interval (CI) values for all 26 variants. For all of the 26 variants we noted that either both or the upper CI value is above ≥ 0.5 kcal/mol, thus confirming the reproducibility of the results (please see details in Supplementary Table 1).

For the affinity-enhancing variants (ΔΔG^Affinity^ scores ≥ 0.5 kcal/mol) from the 13 human proteins (Table 2), we analysed their impact on protein structure and function and their allele frequency distribution related to distinct populations in the gnomAD database and other databases including GenomeAsia 100K (see methods). The structural impact of 26 affinity-enhancing human variants on the local stability of human protein structure was also analysed using Dynamut2 program. The majority of the variants are observed to cause a mild destabilizing impact on the stability on the protein, while only two variants were predicted cause stronger impacts on stability (with < − 2 kcal/mol) (see Supplementary Table 2). However, neither of these are associated with diseases in ClinVar.

According to gnomAD most of the predicted affinity-enhancing variants are rare in human populations, while a few affinity-enhancing variants in IFIH1 and ISG15 are observed to be common (see figure in Supplementary file 3).

For the 13 human proteins, affinity-enhancing variants were analysed in the context of proximity to known and predicted functional sites (Supplementary file 4). The potential mechanisms associated with suppression of the immune system implicated by these variants are discussed in the following section, using some complexes to illustrate our approach.

## Structure–function analyses of predicted affinity-enhancing variants

### Impact of human TOMM70 variants on SARS-CoV-2: ORF9b binding

TOMM70 protein is one of the major human import receptors in the translocase of the outer membrane (TOM) complex. It recognizes and mediates the translocation of mitochondrial preproteins from the cytosol into the mitochondria in a chaperone(HSP90)-dependent manner^105^. It is involved in activation of the innate immune system [GO:0002218] and interacts with ORF9b, which is a key viral innate immune antagonist in SARS-CoV-2^14,105,106^. A study by the Krogan group revealed that TOMM70 is a high-confidence interactor of SARS-CoV-2 ORF9b indicating that binding of ORF9b to the C-terminal domain of TOMM70 is associated with suppression of the innate immune response^33^. We analysed the impact of missense variants using the experimental structure of this complex (PDB ID: 7KDT^14^).

#### Affinity-enhancing variants: structural-function impact and population distribution

**Figure 2:**
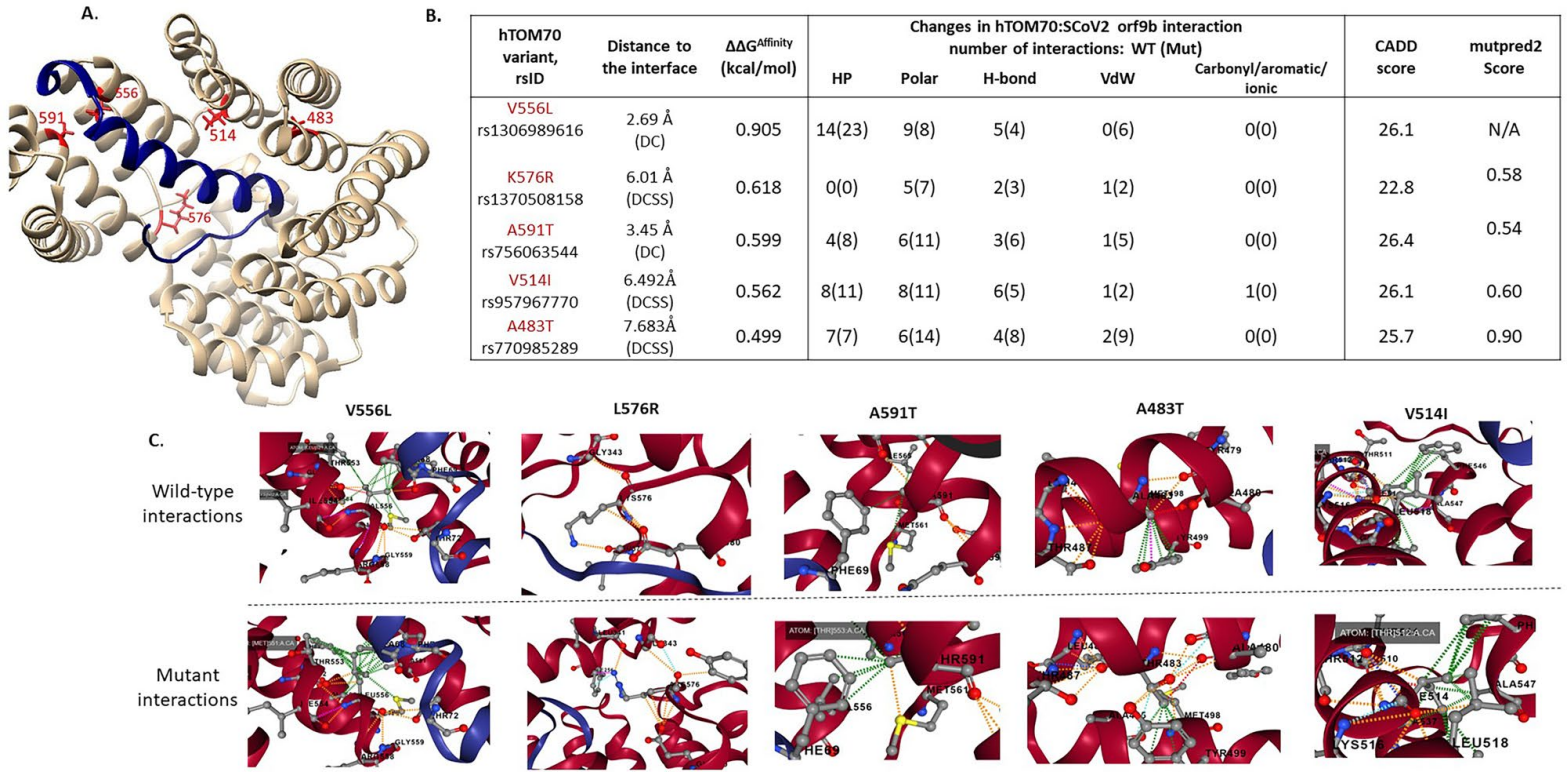
The structure of human TOMM70 in complex with SARS-CoV-2 ORF9b [PDB ID: 7KDT]. (**A**) ORF9b-TOMM70 complex: SARS-CoV-2 ORF9b (blue) interacts with the C-terminal domain of human TOMM70 (tan). ORF9b binds at the substrate-binding pocket in TOMM70. The structural locations of variants (with ΔΔG^Affinity^ ≥ 0.5 kcal/mol) are shown in red. These include three DC-variants (V556L, K576R, A591T) and two DCSS-variants (V514I, A483T). (**B**) The structural impact of DC-variants on atomic interactions at the interface, are shown. HP: Hydrophobic (green), H-bond: hydrogen bond (red), VDW: Van-der-Waals (blue), Polar (orange). (**C**) Effects of affinity-enhancing variants in TOMM70 are shown in detail (figures in 2C source: mCSM-PPI2^55^).

Three DC (directly contacting)-variants in TOMM70 (V556L, K576R, A591T) and two DCSS-variants (V514I, A483T) were predicted to significantly increase affinity (ΔΔG^Affinity^ ≥ 0.5 kcal/mol) (Table 3 and Fig. 2).

**Table 3:** Details of variants in human:TOMM70 that impact binding to SARS-CoV-2:ORF9b.

Variant ID	rsID	Missense variant in gnomAD	mCSM-PPI2-prediction (kcal/mol)	Functional site annotation	GnomAD populations [and allele frequency]
3-100086895-C-T	rs1306989616	p.Val556Leu	0.905	DC_PPI	African/AA [0.00007]
3-100084508-T-C	rs1370508158	p.Lys576Arg	0.618	DC_ PPI	South Asian [0.00003]
3-100084464-C-T	rs756063544	p.Ala591Thr	0.599	DC_PPI	Latino/AA [0.00008] AFR [0.00004] Other [0.0001]
3-100087892-C-T	rs957967770	p.Val514Ile	0.562	DCSS_ PPI	Latino/AA [0.0001] European (non-Finnish [0.00003]
3-100091455-C-T	rs770985289	p.Ala483Thr	0.499	DCSS_ PPI	European (non-Finnish) [0.00002] Latino/AA [0.00002]
3-100092482-A-G	rs1266227924	p.Ile412Thr	− 1.054	DC_ PPI	African/AA [0.00008]
3-100091472-T-C	rs750026792	p.Glu477Gly	− 1.014	DC_ PPI	South Asian [0.000032]
3-100105815-A-G	rs765666545	p.Leu111Pro	− 1.056	near_ligsite	European (non-Finnish) [0.000008]
3-100084470-C-T	rs753612125	p.Asp589Asn	− 0.534	DCSS_PPI	African/AA[0.0000615]; South Asian [0.00003]
3-100086973-C-T	rs775872768	p.Gly530Ser	− 0.545	–	South Asian [0.00003]
3-100093900-C-T	rs762230068	p.Asp397Asn	− 0.536	–	African/AA [0.0001] European (non-Finnish) [0.000008]

For each variant, SNP ID (i.e. rsID), amino acid mutation, mCSM-PPI2 prediction (ΔΔG^Affinity^), functional site annotations and population in gnomAD (in which these variants are present) are indicated. Functional sites are abbreviated as follows- DC_PPI: directly contacting interface site, DCSS_PPI, secondary shell residue from the DC interface site, Ligsite: indicates occurrence in a known ligand binding site and near_ligsite indicates sites that are proximal to a known ligand (HSP90) binding site.

The variant V556L has the highest predicted change in the binding affinity (ΔΔG^Affinity^ score of 0.905 kcal/mol). V556 is a directly contacting (DC) residue at the ORF9b:TOMM70 interface. The wild type V556 in TOMM70 forms hydrophobic bonds with A68 and F69 in ORF9b, and a polar interaction with T72. The mutant V556L gains additional hydrophobic bonds with A68 and F69, resulting in increased predicted affinity for SARS-CoV-2: ORF9b.

The DCSS-variant at residue position 483 lies close to (i.e., within 5 Å) the phosphorylation site in SARS-CoV-2: ORF9b i.e., S53, which is important for binding of ORF9b to TOMM70. The formation of TOMM70:ORF9b complex is regulated via phosphorylation at S53^107^. A483 also interacts with other DC residues in TOMM70. The A483T mutation strengthens this interaction, and this mutation is predicted to be strongly pathogenic by mutpred2 (score = 0.90). Figure 2 summarizes impact of all affinity-enhancing variants and their structural impact on atomic interactions at the interface.

Most affinity-increasing variants in TOMM70 are observed in the African American and American population, but with rare allele frequency i.e., less than 1% (Table 3). Most of the affinity-enhancing variants have an impact on atomic interactions of residues in the interface and are also predicted to have significant impact by various programs including CADD (score > 20), SIFT (predicted as deleterious) and predicted to be pathogenic by mutpred2 (score > 0.5), as shown in Fig. 2B.

In must be noted that African American and Asian populations are also observed to carry affinity-reducing variants in TOM70 that are predicted to decrease binding affinity to SARS-CoV-2:ORF9b (by mCSM-PPI2), thus likely confer resistance to the COVID-19 infection (Table 3). Amongst these, two interface variants (I412T and E477G) are predicted to have strong destabilising impact on binding (< − 1 kcal/mol). Interestingly, the residue E477 is known to be the key residue for ORF9b binding (via binding to Serine 53 in ORF9b [ref.^14^]), thus substantiating the potential effect of E477G variant in abolishing the binding.

### Impact of human IFIH1 variants on SARS-CoV-2: PLPro binding

PLpro (papain-like cysteine protease) in SARS-CoV-2 is involved in a wide range of important functions, such as viral polyprotein chain processing, dysregulation of host inflammatory responses, and impairing the type I interferon (IFN-1) antiviral immune responses^108^. SARS-CoV-2:PLpro plays a key role in innate immune suppression in humans by interacting with various host substrates such as ISG15, IFIH1 and IFIT2, and others^109,110^. Therefore, the SARS-CoV-2:PLpro is a hot spot for designing protein–protein interactor inhibitors^110^.

**Figure 3:**
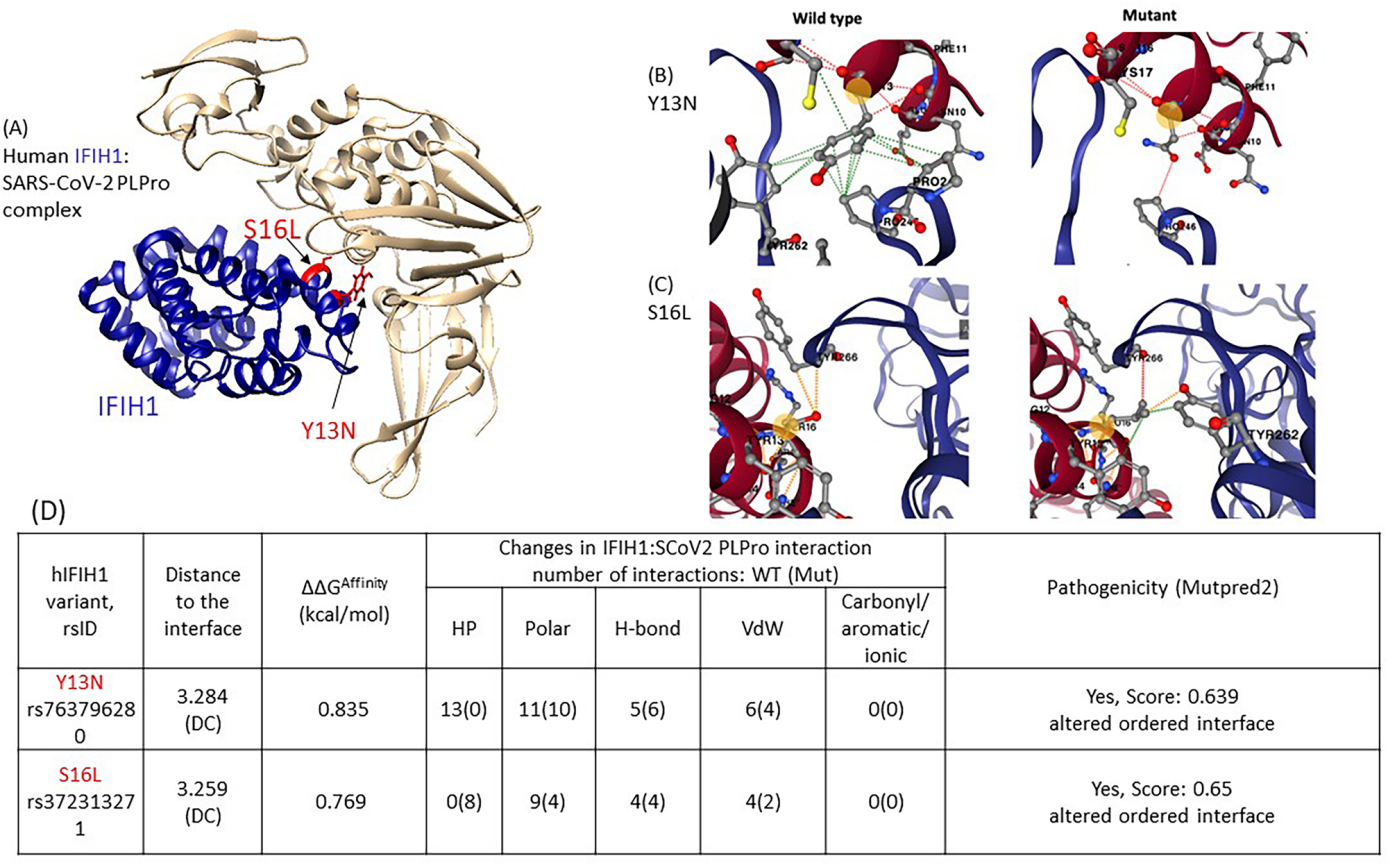
IFIH1-PLpro complex built using AlphaFold2-multimer. (**A**) Human IFIH1 is shown in tan and SARS-CoV-2:PLpro is shown in blue. The structural locations of affinity-enhancing variants (ΔΔG^Affinity^ ≥ 0.5 kcal/mol) are shown in red. These include two DC-variants (S16L, Y13N) (**B**,**C**) Effects of affinity-enhancing variants in TOMM70 are shown in detail (figures in 3B and 3C source: mCSM-PPI2 [55]). HP: Hydrophobic (green), H-bond: hydrogen bond (red), VDW: Van-der-Waals (blue), Polar (orange). (**D**) The structural impact of DC-variants on atomic interactions at the interface, are shown.

IFIH1 (also known as MDA5) is a cytoplasmic innate immune receptor. IFIH1 is a pattern-recognition receptor which binds to viral RNAs and suppresses translation initiation^111^. IFIH1-binding to viral RNA is known to induce type I interferon response by triggering activation of antiviral immunological genes including IFN-alpha, IFN-beta and pro-inflammatory cytokines^111–113^. SARS-COV-2 employs PLpro to bind and block the activation of an IFIH1-dependent cascade of antiviral responses^112^. PLpro is suggested to bind CARD domains of IFIH1^109,112^ and hence we modelled a complex of PLpro and IFIH1 using AlphaFold2-multimer (Fig. 3).

#### Affinity-enhancing variants: structural impact and population distribution

Two affinity-enhancing variants in IFIH1 are predicted—both Y13N and S16L, are DC residues (Fig. 3). According to gnomAD, Y13N is a common variant [allele frequency (AF) > 1%] in East Asians. This is in accordance with GenomeAsia 100K, which indicates Y13N is a common variant in Northeast Asian i.e., in Japanese (AF = 0.01) and Korean populations (AF = 0.003). Upon mutation Y13N, hydrophobic interactions between aromatic rings of Y13 in wild-type IFIH1 and of P245, P246 and Y262 in viral PLpro are replaced by a stronger hydrogen bond between the N13 of the mutant IFIH1 and the P246 of PLpro (Fig. 3).

The other variant significantly impacting SARS-CoV-2:PLpro binding affinity is S16L, observed to occur at a rare frequency in Europeans (non-Finnish). The side chain oxygen atom on residue S16 of IFIH1 forms two weak polar interactions with Y266 of PLpro whereas the leucine side chain in the S16L IFIH1 mutant interacts more strongly with Y266 (Fig. 3). Leucine side chain forms enhanced hydrophobic contacts and polar interaction with neighbouring residues Y262 in PLpro and Y13 in IFIH1 (Fig. 3).

### Impact of human ISG15 variants on SARS-CoV-2: PLPro binding

ISG15 (Interferon stimulated gene 15) plays a key role in the innate immune response to viral infection via a process known as ISGylation upon activation by type I interferons or by viral/bacterial infections. ISGylation (ISG15 modification) is a process whereby ISG15 protein covalently binds to other protein substrates^108,114^. The ISGylation process acts as an antiviral defence mechanism against SARS-CoV-2 and several other RNA viruses. SARS-CoV-2 PLpro binds to ISG15 and blocks the ISGylation^112,114,115^. ISG15 binds to PLpro via the LRGG motif (154–157 residues).

#### Affinity-enhancing variants: structural impact and population distribution

**Figure 4:**
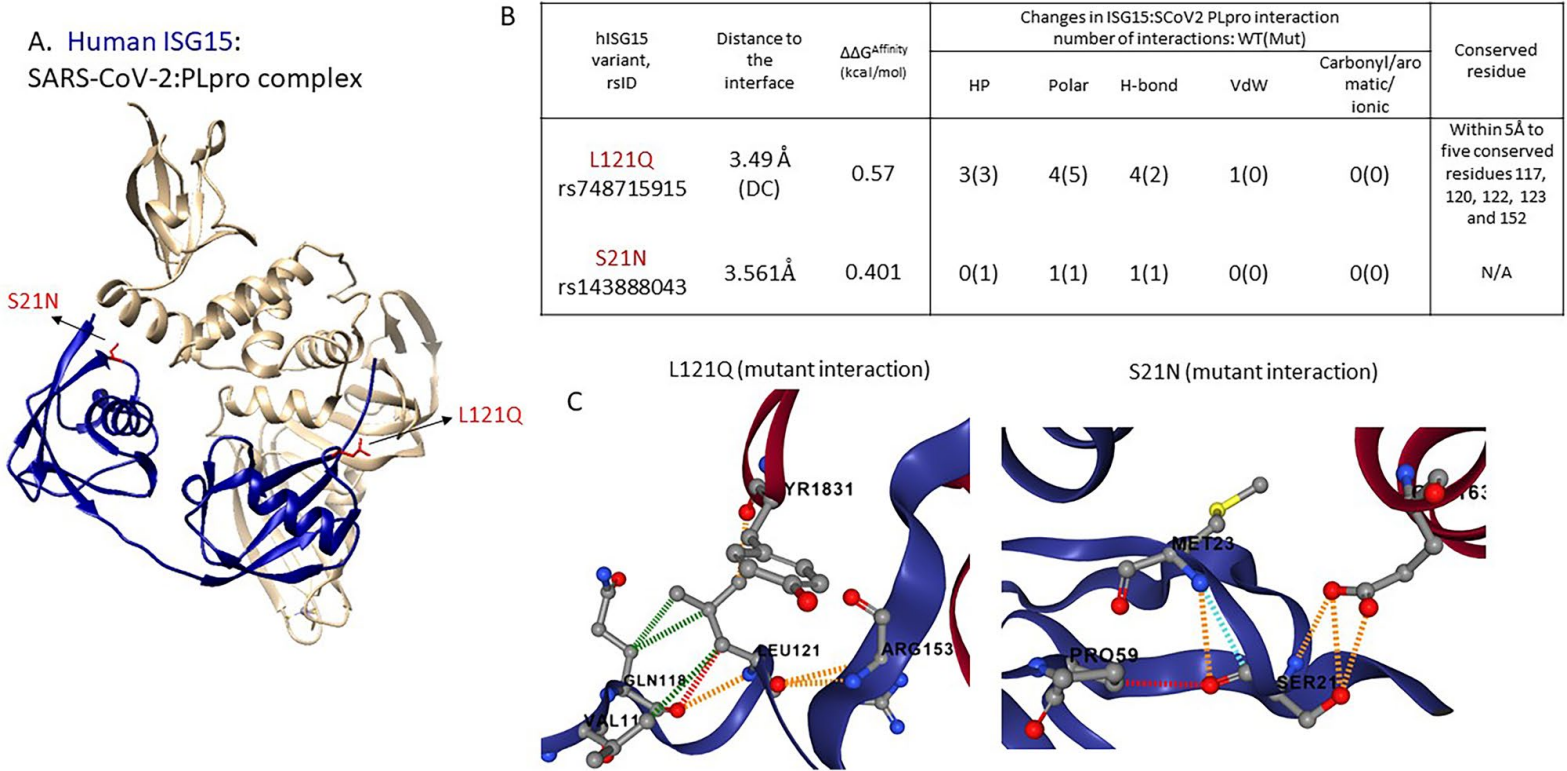
SARS-CoV-2: PLpro-human ISG15 complex (7RBS). (**A**) Human ISG15 (blue) in complex with SARS-CoV-2: PLpro (tan). The affinity-enhancing variants L121N and S21N are indicated in red. (**B**) The table summarizes the impact of affinity-enhancing mutations (wild type vs mutant) on atomic interactions at the interface. Atomic interactions associated with L121Q and S21N are shown in (**C**). HP: Hydrophobic (green), H-bond: hydrogen bond (red), VDW: Van-der-Waals (blue), Polar (orange); Figures in 4(C) are generated using mCSM-PPI2^55^.

Two ISG15 coding variants at DC positions (S21N, L121N) are predicted to enhance the binding affinity of the ISG15-PLpro complex (Table 4, Fig. 4).

**Table 4:** Details of variants in human:ISG15 that impact binding to SARS-CoV-2:PLpro.

rsIDs	Mutation	Mcsm-PPI2 (kcal/mol)	Functional site annotations	Populations in gnomAD and [allele frequency]
rs748715915	p.Leu121Gln	0.57	DC_PPI Near_Scorecons90 Near_LRGG motif	NFE-SWEDISH [0.00003834]
rs143888043	p.Ser21Asn	0.401	DC_PPI	AFR [0.01648] *also supported by GenomeAsia 100K AMR[0.0008475], NFE [0.00007794], SAS[0.00009810], other[0.0008333]
rs1477663018	Leu85Pro	− 1.002	DCSS_PPI, Scorecons90	NFE-North-Western [0.00002404]
rs1160940574	p.Met23Thr	− 1.017	DC_PPI	NFE-North-Western [0.00002387]
rs761507082	p.Arg155Trp	− 1.166	DC_PPI LRGG motif site	AMR[0.0004058] ASJ[0.0001011], SAS[0.00003275],
rs774241776	p.Asn151Lys	− 1.286	DC_PPI Near_ Scorecons90	Other [0.0001647]
rs750338976	p.Arg155Gln	− 2.061	DC_PPI LRGG motif site	NFE[0.00001808]

For each variant, SNP ID, amino acid mutation, mCSM-PPI2 prediction (ΔΔG^Affinity^), functional site annotations and enrichment in specific population in gnomAD are indicated. Functional sites are abbreviated as follows- DC_PPI: directly contacting interface site, DCSS_PPI, secondary shell residue, ligsite: known ligand binding site, Near_ Scorecons90: proximity to conserved sites predicted using Scorecons (score ≥ 0.90).

L121Q, one of the direct contact residues in the interface, is predicted to enhance the ISG15: SCoV2 PLpro binding affinity (mCSM-PPI2 ΔΔG^Affinity^ = 0.57 kcal/mol). This variant occurs within 5 Å from the key LRGG motif (the PLpro recognition site) and forms direct interactions with R153, which is adjacent to this motif, which is therefore likely to have an impact on PLpro-binding. Analyses of proximity to conserved sites detected using Scorecons indicates that the variant lies in the structural neighbourhood (5 Å) of five conserved residues (at positions 117, 120, 122, 123 and 152; with Scorecons90) of which one (W123) is also predicted to be an allosteric site (score: 0.896, predicted using Ohm^116^). This substitution is only observed in the Swedish population at rare frequency.

The second DC-variant S21N is annotated in ClinVar (ID: 475283, benign) and is associated with Mendelian susceptibility to mycobacterial diseases (also known as Immunodeficiency 38 disease)^117^. The variant is associated with severe clinical disease upon infection with weakly virulent mycobacteria (including Mycobacterium bovis and Bacille Calmette-Guerin vaccines)^118,119^. The S21N variant is predicted to moderately increase affinity (with the borderline mCSM-PPI2 ΔΔG^Affinity^ score of 0.401 kcal/mol) and is a common variant (allele frequency > 1%) found in the African population, as supported by multiple population databases including gnomAD [allele frequency (AF): 0.01648], GenomeAsia 100K (allele frequency 0.043269) and All of Us (allele frequency 0.014).

In the case of affinity-reducing (protective) variants (mCSM-PPI2 ΔΔG^Affinity^ ≤ − 0.5 kcal/mol), four variants (R155Q, R155Q, N151D, L85P, M23T) were predicted with significantly reduced binding affinity (ΔΔG^Affinity^ < − 1.0 kcal/mol). Most of these occur only in Non-Finnish Europeans and one predominantly in the American population (R155W). Two variants (R155Q, R155W) occur within the known PLpro-recognition motif in ISG15 (LRGG motif formed by 154–157 residues)^114^. Thus, individuals carrying these mutations may be at lower risk of compromised immunity mediated by ISG15: PLpro binding.

### Impact of human IFIT2 variants on SARS-CoV-2: PLPro binding

**Figure 5:**
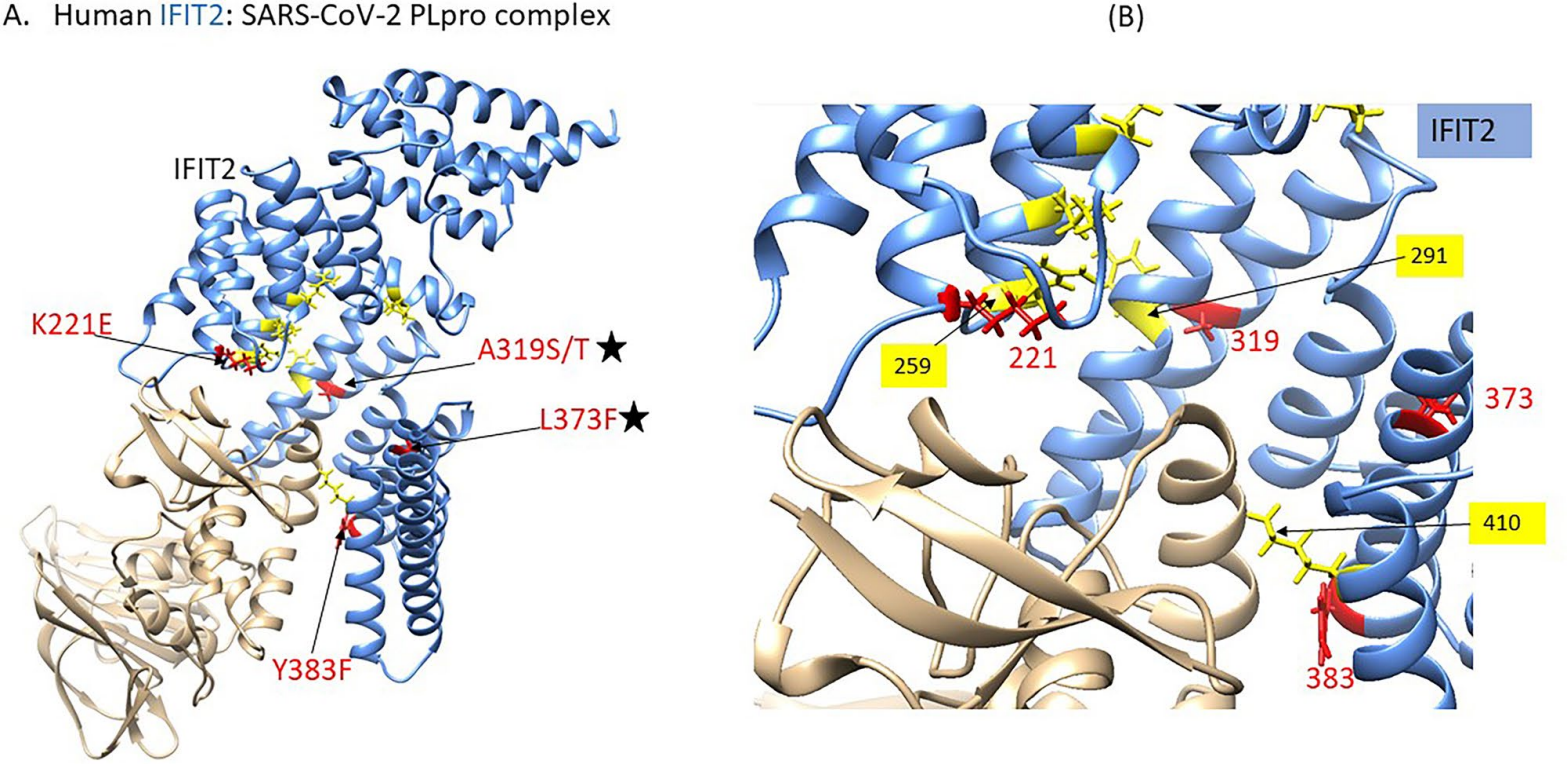
The model of SARS-CoV-2:PLpro in complex with human:IFIT2, and mapping of affinity-enhancing variants (**A**) Mapping of the affinity-enhancing variants (red) K221E, A319S/T, L373F, Y383F onto the IFIT2 (cornflower blue)-SARS-CoV-2:PLpro (tan) complex. (**B**) PLpro interacts with IFIT2 at a site which partially overlaps with a known RNA-binding region (yellow).

IFIT2 (Interferon-induced protein with tetratricopeptide repeats 2) is an RNA-binding protein, and binding of RNA is known to be important for antiviral activity of IFIT2 [127]. Biochemical studies indicate that human IFIT2 binds to SARS-CoV-2:PLpro^109^, however an experimental structure of the complex is not available. The modelled complex in our study, indicates SARS-CoV-2: PLpro binds at a channel-like region at the C-terminus of IFIT2, which is a known RNA-binding region (formed by K37, R184, K255, R259, R291, and K410) [128]. Three PLpro-interacting residues in IFIT2 (i.e., R259, K410 and R291) are involved in RNA-binding (depicted in Fig. 5).

#### Affinity-enhancing variants: structural impact and population distribution

Four IFIT2 DCSS-variants (L373F, K221E, A319S and A319T) significantly affect the binding affinity (mCSM-PPI2 ΔΔG^Affinity^ ≥ 0.5 kcal/mol) (see Table 5). Three of these- A319S, A319T and L373F are also predicted to be pathogenic by CADD (score ≥ 20) and SIFT. Two DCSS-variants in IFIT2 namely, K221E and A319(S/T) are predominant in South Asian and African/AA populations, respectively. The residues (K221E, A319S and A319T and Y383F) directly interact with residues (R259 and R291) known to be involved in the RNA-binding in IFIT2 (Fig. 5B). Thus, mutations in IFIT2 protein which increase binding to SARS-CoV-2:PLpro are likely to hinder the binding of IFIT2 to RNA, and thus the normal antiviral mechanism of IFIT2.

**Table 5:** Details of affinity-enhancing variants in human IFIT2 (SARS:CoV:2-PLpro interacting protein).

Variant ID	rsID	Mutation	mCSM-PPI2 (kcal/mol)	Functional site annotations	Populations in gnomAD [ Allele frequency]
10-91066830-C-T	rs761596604	p.Leu373Phe	0.911	DCSS_PPI	South Asian [0.00003268] Asian: 0.00004 (All of Us)
10-91066374-A-G	rs1305223950	p.Lys221Glu	0.616	DCSS_PPI Near_ligsite Near_Scorecons90	South Asian [0.00003268] Asian: 0.00002 (All of Us)
10-91066668-G-T	rs775027680	p.Ala319Ser	0.532	DCSS_PPI Near_ligsite Near_Scorecons90	African/African American [0.00006460]
10-91066668-G-T	rs775027680	p.Ala319Thr	0.516	DCSS_PPI Near_ligsite Near_ Scorecons90	African/African American [0.00006460] East Asian: 0.00007130 Latino/Admixed American [0.00007008] Swedish [0.00003832]
10-91066861-A-T	rs751422356	p.Tyr383Phe	0.449	Near_ligsite	European NFE [0.00001556]

For each variant, SNP ID, amino acid mutation, mCSM-PPI2 prediction, functional site annotations and enrichment in specific population in gnomAD are indicated. Functional sites are abbreviated as follows- DC_PPI: means directly contacting interface site, DCSS_PPI, secondary shell residue, ligsite: indicates known ligand binding site (i.e. RNA binding site in IFIT2) and near_ligsite indicates sites that are proximal to a known ligand binding site. near_Scorecons90: proximity to conserved sites predicted using Scorecons (score > 0.90).

Thus, our analyses of 3D complexes, affinity-enhancing variants, and functional sites identifies some affinity enhancing variants that could promote the binding of host immune proteins to SARS-CoV-2 proteins, thereby reducing the binding to their natural protein partners. This could mediate reduced immune responses in certain individuals.

### Impact of variants in SARS-CoV-2 proteins

In addition to human protein variants, we also analysed the impact of viral protein variants, in Spike-RBD, Spike-NTD, PLPro, ORF9b, ORF7b, ORF6, ORF3a, NSP7, NSP15, NSP14, NSP1, N and E (using experimental/predicted complexes listed in Table 1). The impact of a total of 212 viral variants occurring at protein–protein interface region was analysed using mCSM-PPI2. The majority of the variants are observed to have ‘moderate’ impact on binding to their human interactors, i.e. predicted binding affinity (ΔΔG^Affinity^) values range from 0 to 0.49 kcal/mol for affinity-enhancing variants (see supplementary Table 3).

However, a few variants in SARS-CoV-2 proteins such as NSP14 (L6074F and N6054I), PLpro (L1774F) and Spike (S477N) are significantly predicted to be affinity-enhancing (mCSM-PPI2 ΔΔG^Affinity^ ≥ 0.5 kcal/mol). The impact of the variant spike-RBD S477N on enhanced ACE2-binding affinity is recently confirmed by experimental assays^37^. Among the five VOC’s Omicron has evolved to contain highest number of mutations in the spike protein. The phylogenetic tree of all VOC’s and VOI’s was built from the Spike protein sequences, to better understand the pattern of emergence of Omicron and Omicron subvariants (Please see phylogenetic tree in Supplementary Fig. 1). Interestingly, a comprehensive study by Martin et al. suggest that S477N is common in all Omicron sub-variants and is under positive selection pressure^120^. The impact on binding affinity to the ACE2 receptor, thus may provide a selective advantage to increase transmissibility in the host.

### Pathway enrichment analyses

We examined the biological processes and signalling pathways associated with the 13 human proteins containing affinity-enhancing variants (Table 2) using three well-established pathway enrichment databases: Gene Ontology (GO), KEGG and Reactome (Table 6). The most significantly enriched terms were related to immune functions, including viral life cycle (GO biological process) and Influenza A (KEGG). The top enriched Reactome pathway was SARS-CoV-2 Infection, confirming the association with this panel of proteins identified in earlier analyses.

**Table 6:** Top significant GO, KEGG^98^ and Reactome pathways associated with 13 SARS-CoV-2-interacting human proteins (containing predicted affinity-enhancing variants).

Term	Pathway	Adj. p-value
REAC:R-HSA-9694516	SARS-CoV-2 infection	2.59 × 10^–8^
GO:1903900	Regulation of viral life cycle	3.19 × 10^–5^
KEGG:05164	Influenza A	0.0019

Since many biological processes are governed by functional modules which are highly interconnected sub-networks of protein–protein interactions, identifying functional modules containing the 13 human proteins provides a method for gaining further insights into their biological functions. This approach can also offer insights into specific mechanisms contributing to SARS-CoV-2 pathology.

To this end, we used two state-of-the-art modularity detection algorithms and applied them to two well regarded network datasets: STRING, and ConsensusPathDB. We then identified which modules contain any of the 13 proteins and performed pathway enrichment analysis on each of the modules using GO (biological processes), KEGG and Reactome. The enrichment analysis was used to assign to each of the 13 proteins the appropriate biological processes/pathways. For a full list of proteins in each module, see Supplementary files 5 and 6. We constructed a protein–protein interaction network for all these human proteins and their associated partner viral proteins (please see Supplementary file 7).

Unsurprisingly, many of the 13 proteins were associated with immune response to viral infection, including interferon-inducible genes IFIT2 and IFIH1, and ISG15 involved in modulating viral replication. Induction by type I interferons (α/β), which activate other immune cells, was also associated with the transmembrane protein TRIM25.

Several symptoms, particularly in severe or long COVID-19 cases, are associated with mitochondrial dysfunction, including cytokine storms^121^, which is a key pathway associated with TOMM70 associated with mitochondrion organisation. It has also been found to be involved in interferon regulation^122^. SARS-CoV-2 viral proteins replicate in the cytoplasm via translation on ribosomes, hence supporting our identification of several pathways involved in cytoplasmic or ribosomal transport, specifically NUP98, RAE1, ribosomal protein RPS3, TOMM70 and TRIM25. Other genes are involved in cell processes that are hijacked by the virus during infection. For example, ARF6 was identified as involved in exocytosis, but is used by the virus to infiltrate and infect the cell^123^. Overall, the findings of our functional analysis highlight some key mechanisms involved in the viral response to COVID-19.

### Functional families associated with small molecule inhibitors

For the 13 human proteins containing predicted affinity-enhancing variants, we analysed their associated functional families in CATH (CATH-FunFams) to inspect whether any of the homologous proteins in the FunFam were linked to known small molecule inhibitors. These small molecules, which may include drug-like molecules or approved drugs, were identified using the ChEMBL database (see Table 7).

**Table 7:** Human proteins with CATH-FunFams containing small molecule inhibitors.

Protein	CATH superfamily and functional family (CATH-FunFam) ID	Associated entry in ChEMBL	Representative 3D-structure
IFIH1	1.10.533.10 CATH-FunFam ID 77	CHEMBL4739862 [CHEMBL4594258, phase 2]	7DNI [PDB]
AXL	2.60.40.10 CATH-FunFam ID 810	CHEMBL4895 [CHEMBL3301622, approved] CHEMBL4879451	5VXZ [PDB]
ARF6	3.40.50.300 CATH-FunFam ID 286	CHEMBL5987 CHEMBL1075274	3LVR [PDB]

Three human proteins (IFIH1, AXL and ARF6) are observed to be linked with small molecule inhibitors from ChEMBL. The associated entries in ChEMBL are indicated. The representative 3D structure for each functional families in CATH is indicated. If the representative is not available in CATH, the representative from PDB is shown.

Next, we used CavityPlus to detect druggability of the protein–protein interface formed by these three proteins. CavityPlus provided strong confidence for prediction for druggability of IFIH1: PLpro interface and ARF6:NSP15-interface, and medium confidence for AXL:NTD interface (see supplementary file 8). These observations are also supported by the CASTp prediction (see Supplementary file 9). These results are indicative of potential applications of their associated inhibitors for designing drugs targeting these protein–protein interactions.

For example, IFIH1 is associated with Selgantolimod in ChEMBL (ID: CHEMBL4594258), which is in phase 2 clinical trial. Selgantolimod is known to be a Toll Like Receptor 8 Agonist, which increases immune responses in chronic Hepatitis B patients^124^. Analyses using CavityPlus provided a strong prediction score for the presence of two cavities that occur at the IFIH1-PLpro interface (the topmost cavity with score of 4177.0, Supplementary File 8). These cavities do not interfere with CARD oligomerization or its protein partner MAVS, which is required for IFIH1- induced interferon response, thus substantiating the potential use of this molecule in designing therapeutics against SARS-CoV-2 infection. Docking of this ligand with IFIH1-PLpro, provides support for binding at this cavity (^125^; see Supplementary File 10).

Likewise, cavities in the interfaces of NTD-AXL and ARF6-NSP15 could likely be interesting targets for drug design and further experimental assays are required to substantiate their application for drug repurposing or as starting points for structure-based design of novel compounds.

## Discussion

We analysed ~ 20% of SARS-CoV-2 immunity associated interactions using structural data of the protein–protein complexes. Structural analyses helped in detecting affinity-enhancing variants in human immunity-associated proteins and in evaluating their possible impact on SARS-CoV-2:human protein complexes and COVID-19 susceptibility. Information on the proximity of these variants to functional sites in the 3D structure can give insights into potential mechanisms associated with the suppression of normal functioning of these immune proteins, thereby affecting COVID-19 susceptibility.

We applied a structural bioinformatics approach to analyse the impact of missense variants from human and viral proteins, using 19 SARS-CoV-2: human protein structural complexes (obtained from PDB or built using AlphaFold2-multimer/ptm). We analysed 468 coding variants in human proteins occurring at protein–protein interfaces. A total of 26 affinity-enhancing variants from 13 human proteins were predicted to significantly enhance SARS-CoV-2 binding.

A majority of these human proteins were involved in key immune pathways and associated with antiviral activity against SARS-CoV-2, including Interferon stimulating genes (ISG15, IFIT2); important receptors (such as IFIH1, TOMM70); proteins involved in nucleocytoplasmic shuttling of viral mRNA (NUP98 and RAE1), proteins involved in cellular translation machinery (RPS2 and RPS3) and cell entry receptors via spike-binding (ACE2, KREMEN1 and AXL). Among these 13 proteins, experimental assays have been performed on spike-ACE2, substantiating the role of the predicted spike affinity-enhancing variants in COVID-19 susceptibility and transmission^37,38^, while variants in the remaining proteins are reported for the first time in this study.

The modelling of complexes using AlphaFold2-multimer/ptm increased the structural coverage of the complexes and helped to provide structural insights into the mechanisms of SARS-CoV-2 proteins binding to human host proteins. We propose that affinity-enhancing variants in key-immunity associated human proteins could promote their binding to SARS-CoV-2 proteins, competing with human protein partners or substrates in immune pathways, and this in turn, may have an impact on COVID-19 susceptibility. This finding is in line with previous experimental studies on SARS-CoV-2: human interactions that affect natural immune pathways^126,127^. For example, Li et al.^127^ suggest that binding of SARS-CoV-2: ORF6 to human: NUP98-RAE1 complex competitively inhibits mRNA binding (to NUP98-RAE1), which is essential for its immune function. Likewise, overexpression of SARS-CoV-2: Nucleoprotein protein is observed to be associated with the attenuation of RIG-I-mediated interferon production via binding to TRIM25 and thus interrupting the interaction between TRIM25 and RIG-I (which is its natural protein partner)^126^.

The SARS-CoV-2:human protein interactions associated with these 13 human proteins could be attractive targets for drug design that target the protein–protein interfaces. In particular, SARS-CoV-2: PLpro is known to be a promising target for designing protein–protein interaction inhibitors^128^. In our analyses, we modelled SARS-CoV-2:PLpro interactions with ISG15, IFIH1 and IFIT2. Interestingly, PLpro interactor protein IFIH1 is associated with drug-associated functional family in CATH. Likewise, we observed druggable CATH-FunFam for AXL and ARF6 proteins (interacting with Spike-NTD and NSP15 in SARS-CoV-2, respectively). The domain relatives within CATH-FunFams exhibit highly conserved drug binding sites and have the potential to be the druggable entities within drug targets, as shown in^60^. Thus, further studies targeting such immune proteins and PLpro-mediated interactions would be helpful.

We suggest monitoring both common and rare variants in human proteins, that are predicted to cause significant impact and thus likely to be associated with disease pathogenicity or susceptibility. Two affinity-enhancing variants – one from IFIH1 (Y13N) and one from ISG15 (S21N), are observed to occur at > 1% allele frequency i.e. common in East Asian and African populations, respectively. These affinity-enhancing variants exhibit distinct allele frequency distributions across gnomAD populations and thus it is likely that they exhibit distinct COVID-19 susceptibility. For example, the variant Y13N in IFIH1 is common mutation found only in East Asians. The S21N in ISG15 is common in African population and less common in other populations such as Middle Eastern, Admixed American, European (non-Finnish) populations and not present in any other populations in gnomAD. Thus, affinity-enhancing variants involved in increased affinity to SARS-CoV-2 binding, could manifest population-scale differences in causing susceptibility to the disease via alteration to natural immune pathways in humans.

Most of the variants identified in our study have allele frequencies (< 1%). A growing number of studies support the key role of rare variants in causing susceptibility/severity to COVID-19^12,26–32^. Thus, apart from common variants in East Asian and African population, rare variants predicted from various other populations are predicted to be affinity-enhancing, which could affect COVID-19 susceptibility and cause increased risk of infection in individuals carrying them.

We also provide a catalogue of affinity-reducing variants from 12 proteins (Table 2), particularly in ISG15 and TOMM70. These variants are observed to significantly reduce binding affinities of the human proteins to their SARS-CoV-2 partners and some of which are observed to occur at key functional sites/motif region that are important for binding to SARS-CoV-2 proteins, thus their likely role in resistance to COVID-19. Earlier data showed a good correlation between predicted affinity-reducing variants and experimental observations^38^. Thus, affinity-reducing variants reported in this study could provide an explanation for why some individuals in specific populations are less likely to experience SARS-CoV-2 associated immune evasion.

Whilst we observed only two common variants in certain specific ethnic groups, occurrence of certain rare affinity-enhancing variants could also lead to increased susceptibility in individuals carrying them. Though the data suggest some role of genetic variation in COVID-19 susceptibility, the role of social and environmental factors should also be studied. In addition, our study is based on computational prediction of changes in the binding affinity. Experimental data is available for variants in only a limited number of complexes such as spike-ACE2 and ORF9b-TOMM70. Future experimental studies would be helpful for validating the impacts of proposed affinity-enhancing mutations in other immune proteins.

## Conclusions

We used structural bioinformatics approaches to predict human and viral protein variants affecting COVID-19 susceptibility and to suggest repurposing of therapeutics based on CATH functional family data associated with small molecules/drugs. A total of 26 affinity-enhancing variants are reported in our study and we discuss their structural impact in the context of functional sites using 3D structures of SARS-CoV-2: human complexes. The protocol designed in this study could be extended to analyse other protein interactions as more structures are experimentally determined and more powerful tools for protein structure prediction emerge. Our approach could be helpful in future studies not only for COVID-19 but also other emerging infectious diseases.

### Supplementary Information

Supplementary Information.

## Data Availability

The data supporting the conclusions of the study is made available in the Supplementary files 1 to 8. The dataset of SARS-CoV-2:Human 3D-complexes used and generated in this study is provided in Supplementary file 9. The datasets generated and/or analysed during the current study (.pdb files for 3D-complexes and associated accessions) are available in the Zenodo repository, web link https://zenodo.org/records/10090696.
