# Supplementary material for: Predicting human and viral protein variants affecting COVID-19 susceptibility and repurposing therapeutics
Source: Sci Rep. 2024 Jun 20;14:14208. doi: 10.1038/s41598-024-61541-1 (PMC11190248; doi:10.1038/s41598-024-61541-1)
Supplement: Supplementary file 1 — Supplementary Information. [file 41598_2024_61541_MOESM1_ESM.zip › Supplementary files(allincludingrevised)_13May_2024/Supplementary file 10-dockingIFIH1-plro.docx]

**Supplementary File 10**

**Docking of IFIH1:PLpro complex with ligand with Selgantolimod** (ChEMBL ID: CHEMBL4594258)

**Figure: Docking of** **Selgantolimod with IFIH1:PLpro complex. The ligand binds at the cavity formed by IFIH1:PLpro interface.**

1. Docking of Selgantolimod (blue) with IFIH1 (green) :PLpro (orange) is performed using AutoDock and Autodock Vina. (AutoDock Vina score: -7.4).


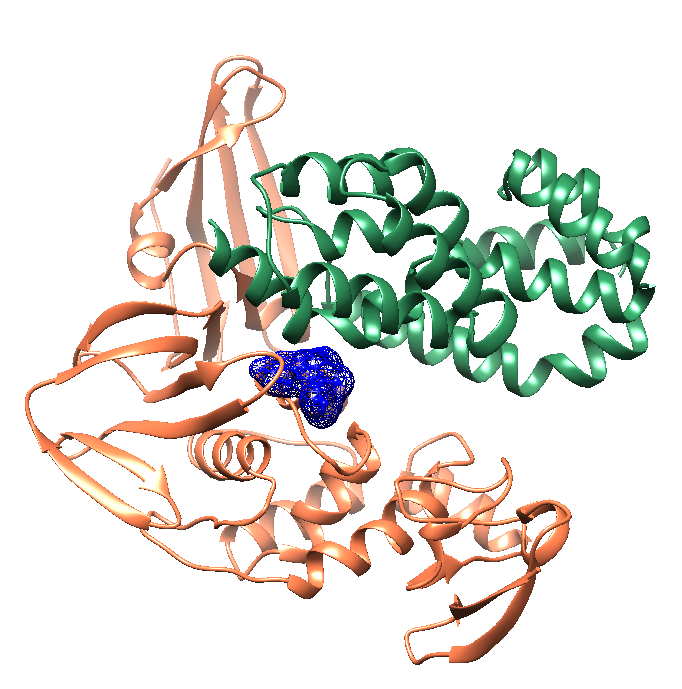


**(B) Selgantolimod binds at the cavity strongly predicted by Cavityplus (score: 4177.0). The cavity residues are shown is purple.**


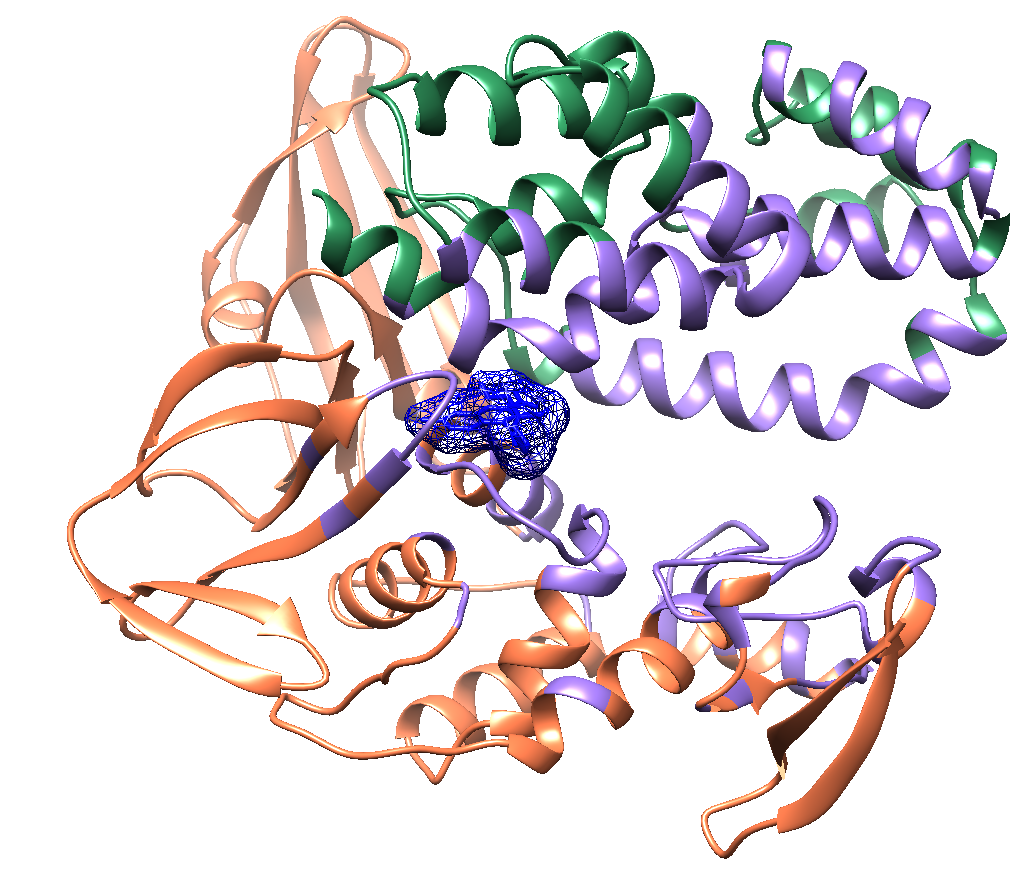

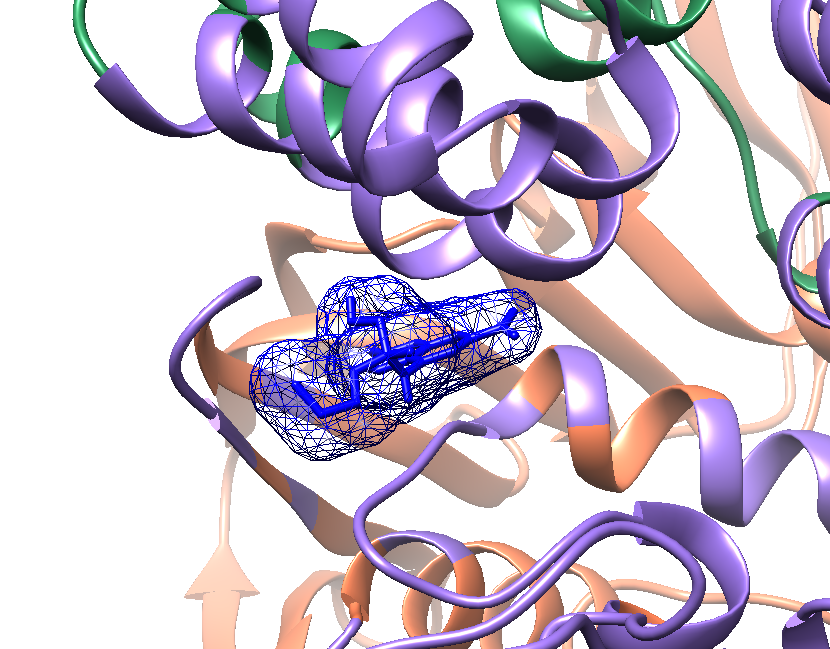


AutoDock references:

Morris, G. M., Huey, R., Lindstrom, W., Sanner, M. F., Belew, R. K., Goodsell, D. S. and Olson, A. J. (2009) Autodock4 and AutoDockTools4: automated docking with selective receptor flexiblity. J. Computational Chemistry 2009, 16: 2785-91.

Goodsell, D. S. and Olson, A. J. (1990), Automated Docking of Substrates to Proteins by Simulated Annealing Proteins:Structure, Function and Genetics., 8: 195-202.

ChEMBL reference:

Mendez, D et al (2019) ChEMBL: towards direct deposition of bioassay data. Nucl. Acids Res, 47, D930-40
