# Supplementary material for: Predicting human and viral protein variants affecting COVID-19 susceptibility and repurposing therapeutics
Source: Sci Rep. 2024 Jun 20;14:14208. doi: 10.1038/s41598-024-61541-1 (PMC11190248; doi:10.1038/s41598-024-61541-1)
Supplement: Supplementary file 1 — Supplementary Information. [file 41598_2024_61541_MOESM1_ESM.zip › Supplementary files(allincludingrevised)_13May_2024/Supplementary file 5-modules-covid-cpdb.docx]

**Supplementary file 5** :

ConsensusPathDB modules (COVID-19 human genes)

13 RPL24 RPL7 RC3H2 AGO1 RPL37A RPL21 RPL14 RPL3 RPL36 VRK1 RPS20 RPS25 RPS19 RPL10 RPL12 RPS10 RPL7A RPS2 RPL35A RPL30 RPS15A RPSA CEP250 RPL15 RPL31 RPS27A RPLP2 EEF1A1 RPL27 PAN2 RPLP1 RPL22 RPL13 RC3H1 RPS4X RPS27L RPL10L RPL10A RPS7 RPS12 RPL11 UBL4A RPS5 RPS6KB2 RPL18A RPL8 TRIM25 RPL23A RPL35 RPL29 RPL3L FBP1 AGO2 RPL9 RPL38 HMGB1 RPS27 RPS26 RPS3A INS REST MAP3K14 LARP7 RPL34 RPS23 RPL27A RPS11 ARIH2 RPLP0 RPS3 RPL4 RPL13A

33 GPD2 IMMT HTRA2 TIMM13 TIMM8A IMMP2L COA6 OMA1 IMMP1L TIMM9 ENDOG TIMMDC1 TTC19 APOO COX5B COX4I1 OPA1 AFG3L2 SFXN1 MICU2 TIMM8B CPOX TOMM70 CHCHD3 USP30 AIFM1 DIABLO TDRKH MICOS13 COX6B1 PARL SLC25A13 MAVS DNAJC11 PLGRKT APOOL SAMM50 SLC25A12 YME1L1 TIMM10 MICU1 OCIAD1 MTX2 TOMM20 LACTB MTCH1 MTX3 HCCS COX14 SCO1 FAM136A COX15 SLC25A51 CISD1 COX5A MTX1

40 WNT5A FZD4 ICOSLG ICOS FZD7 WNT4 LRP6 DKK1 WNT3A FZD1 LRP5 WIF1 WNT1 WNT2 FZD9 SFRP1 FZD6 FZD8 WNT7A FZD5 RSPO1 KREMEN1 TSKU AQP5 WNT2B FSTL1 AFM RSPO2 SCGB1D2 PORCN WNT9A NKD1 MELTF SFRP2 WNT11 KRTAP5-8 ZNRF3 WNT5B WNT10B RNF43 WNT7B UQCR11 APCDD1 NOTUM WNT10A CAPRIN2 WNT8A RSPO3 GPC3 MT-CYB WNT6 WNT16 LGR5 SFRP5

102 NUP93 RANBP2 LMNA NUP153 KPNA1 NUP43 NUP188 TPR RAE1 AHCTF1 KPNB1 NXF1 NUP205 NUP107 NKX2-1 KPNA2 NUPR1 KPNA3 NUP98 NUP62 NUP160 SEH1L MYC SEC13 NUP155 KPNA4 NUP214 NUP85 RCC1 NUP133 SENP2 RAN NUP50 RANGAP1 IPO5

132 DCC UNC5A NTN1 NEO1 UNC5B DSCAM NTN3 NELFB UNC5C NELFCD EIF2B5 C1orf50 EIF2B1 TPRX1 TRIML2 NTN4 TRIM9 DNAAF4 TRIM46 NELFA EIF2B4 FKBP3 EIF2B2 FLRT3 TENM2 DRAXIN ADGRL3 EIF2B3 ADORA2B DENR

162 LGALS3 PRDX1 LDHB TKT TXN AGR2 PINK1 ENO1 ISG15 DSTN CFL1 PRDX2 GSTP1 EEF2 TPI1 SOD1 ACO2 TFCP2 PRDX6 PPIA WDR1 ZFP36L2 YAP1 GAPDH PKM ALDOA PGK1 PARK7

186 GAST PSG1 NTSR1 CPE SECTM1 PCOLCE2 ACE2 NTS SLC6A19 NPC2 TMPRSS11D PSG3 SECISBP2L APLN PSG5 PSG11 PSG9 LOXL2 SDF2 PSG4 PSG6 NTSR2 TMPRSS11A KLK11 PSG8 CSH1

293 DDX58 IFIH1 IRF8 TBKBP1 DHX58 AZI2 RNF125 IRF6 NLRC5 RNF135 ZFAND6 FNDC11 TLX2 PNMA6A RNF122 RIMBP2 BNC2 ZCCHC3 USP3

338 RNF25 STING1 S100A7 IFIT1 NKD2 IFIT3 DDX3Y ELMOD3 LVRN IFIT2 DHX40 IFIT1B METTL23 PPP1R14C PHACTR4 SAP25 IFNA10

496 PRPF8 ANXA2 CACYBP RUVBL1 RUVBL2 PEBP1 EIF6 ARF6 HLA-B

526 OSTF1 TYRO3 CD44 ERRFI1 AXL FGFR4 SHC3 GRM1
