# Supplementary material for: Predicting human and viral protein variants affecting COVID-19 susceptibility and repurposing therapeutics
Source: Sci Rep. 2024 Jun 20;14:14208. doi: 10.1038/s41598-024-61541-1 (PMC11190248; doi:10.1038/s41598-024-61541-1)
Supplement: Supplementary file 1 — Supplementary Information. [file 41598_2024_61541_MOESM1_ESM.zip › Supplementary files(allincludingrevised)_13May_2024/Supplementary file 6-modules-covid-string.docx]

**Supplementary file 6:**

STRING modules (COVID-19 human genes)

16 EIF2S1 RPS18 HBA2 EIF1AD EIF3M EIF3C RPS16 RPSA RPS2 RPS3 EIF3L NOB1 EIF1AX RPS3A RACK1 EIF5 ABCE1 EIF3G RPS8 RPS23 CSNK1E RPS24 ENSP00000346046 RPS10 EIF3D RIOK1 RPS27L RPS15 RPS15A CSNK1D EIF2S3 GSPT1 EIF3J RPS11 TSR1 EIF3CL EIF3H RPS19 EIF3B RPS29 RPS4X RPS25 LTV1 RPS21 EIF1 RPS9 RPS13 RPS20 EIF3I HBA1 EIF3A RPS28 MCTS1 EIF1B EIF2A ENSP00000357555 EIF4A1 EIF3F RPS14 EIF3K LRRC47 EIF2S2 RPS5 RPS26 EDF1 RPS12 EIF4G1 DHX29 RPS6 DENR RIOK2 EIF2D EIF3E FAU

31 MSN PRKCI PARD6A NOTCH2 LLGL2 NOTCH2NLR JAG1 ENSP00000277541 LLGL1 PARD6G CRB2 RASSF10 PARD3 PARD6B JAG2 PARD3B CRB1 NOTCH3 ENSP00000464060 MIB1 LRRC1 DLL1 BTBD8 EGFL7 ENSP00000384136 GPSM2 MAML1 PALS1 RASSF7 PPP1R13B RASSF8 NEURL1B NEURL1 DLL3 ECT2L TACC3 MAML3 CRB3 INSC TP53BP2 NUMBL MCF2 EPB41L4B DTX2 LFNG NOTCH4 DNER DTX1 DLL4 MAMLD1 MAML2 ELF3 DTX4 CNTN1 FRMD6 FRMPD4 NRARP KIAA1614 ELF4 MFNG RFNG POFUT1 PTCRA LAYN

59 EXOC7 RAB11FIP4 ASAP1 ASAP3 RAB11FIP3 ARF6 USP6 RHOQ RALA EXOC5 EXOC8 EXOC2 TNFAIP2 EXOC6 EXOC1 EXOC3 EXOC4 RALB EXOC6B STXBP6 RUBCN OAS1 EXOC3L4 EXOC3L1 EXOC3L2 ACAP1 GULP1 ENSP00000377140 CNGA2 CNGA1 PKD1 TRIP10 ENSP00000293970 SLC17A6 CNGB1 PKD2 MEGF6 FUT2 CNGA4 ENSG00000260371 LAPTM4B CNGB3 FAM3D PKHD1 PKD1L3 PKD1L1 ENSP00000485172 ENSP00000485105 MEGF10 SLC24A1 PKD2L1 SLC24A2

99 ZC3H12A SPATA2 IKBKE RNF11 ZBP1 DHX58 IRF7 IKBKB TRAF3IP2 TRIM25 OTULIN STING1 RNF31 IRF3 TNFAIP3 MAVS TBKBP1 TRIM14 RNF135 IKBKG ENSP00000263642 TNIP3 CYLD ENSP00000480499 OPTN PYDC1 NLRX1 TANK SHARPIN SASH1 TBK1 AZI2 NLRC5 RBCK1 MAP3K1 TRIM40 CHUK TAX1BP1 NLRP4 DDX58 TNIP1

102 TXNDC5 HPS5 GPRC5A TRIM69 DTNBP1 KXD1 TSKS SLC14A2 BLOC1S6 BLOC1S5 SNAPIN ENSG00000258311 HPS6 HPS4 HPS1 BLOC1S3 BLOC1S4 HPS3 BLOC1S2 BLOC1S1 BCAS4 CCDC57 ODF2L SPATS2 PEAR1 TCL1B TRIML2 C20orf202 BORCS6 TEX35 BORCS5 ZNF706 CMYA5 BORCS8 EBAG9 BORCS7 CALHM4 DBNDD2 CCDC185 CCDC122

138 NUP210L NUP188 RANGAP1 NUP210 NUP93 NUP85 NUP214 NXF1 TMEM209 NUP42 NUP155 NUP37 RAE1 NUP43 NXT1 NUP107 NDC1 NUP35 POM121 NUP62 TPR NUP88 NUP98 POM121C NUP54 SEH1L AAAS NUP205 NUP133 NUP160 NUP50 RANBP2 NUP58 NKX2-1 AHCTF1

164 PTK2 ENSP00000478570 HGF NCK1 SRC PDGFB ELMO1 NCK2 CRK KDR PTPN12 DAB1 DOCK1 FLT4 ABL2 CRKL ABL1 SHB AXL ELMO2 NEDD9 PDGFRA RAPGEF1 PTK2B SH2D2A PDGFC FLT1 PDGFRB BCAR1 MET PDGFA

178 MFN1 CHCHD10 TOMM40L TOMM7 CHCHD4 MICOS13 CHCHD2 MICOS10 TOMM70 SLC25A46 SAMM50 TOMM20 MTX2 ANKZF1 APOOL APOO TMEM11 CHCHD6 CHCHD3 TOMM40 SFXN1 TOMM20L TOMM5 DNAJC11 MTX1 TOMM22 MTX3 IMMT TOMM6

200 LRP6 RNF43 LGR5 RSPO4 RSPO3 LGR4 LRP5 TSPAN12 KREMEN2 ADGRA2 DKK2 CAPRIN2 ZNRF3 LGR6 KREMEN1 MESD RSPO1 DKK4 LRP4 RECK ENSP00000482378 SOST DKK3 RSPO2 DKK1 ENSP00000367301

210 DEFA3 ERAP1 ACE2 CLTRN ENSG00000286088 DEFA4 DEFA1 DEFA1B DEFA5 DEFA6 BACE2 TMPRSS2 NUCB2 CTSF TRHDE ERAP2 SLC6A20 SLC6A19 SLC6A18 MRGPRD TMPRSS11D ENSG00000273171 MRGPRE SLC36A2

211 IFIT3 DNAAF2 PIH1D2 LONRF3 IFIT2 TMTC3 SPAG1 IFIT1B TTC16 IFIT1 TMTC4 TMTC2 TTC32 TTC24 IFIT5 CUTC CFAP52 PHACTR4 SAP25 CFAP300 NADSYN1 TMEM183A LVRN PPP1R14C
