# Supplementary material for: Predicting human and viral protein variants affecting COVID-19 susceptibility and repurposing therapeutics
Source: Sci Rep. 2024 Jun 20;14:14208. doi: 10.1038/s41598-024-61541-1 (PMC11190248; doi:10.1038/s41598-024-61541-1)
Supplement: Supplementary file 1 — Supplementary Information. [file 41598_2024_61541_MOESM1_ESM.zip › Supplementary files(allincludingrevised)_13May_2024/Supplementary File 7 - PPI network.docx]

**Supplementary File 7 : Protein-protein interaction network of SARS-CoV-2:Human protein interactors**

**Supplementary File 7**

Protein-protein interaction network showing SARS-CoV-2 proteins (red triangles), human interactors (blue circles) and human module proteins derived from Consensus PathDB (yellow circles).

Module functions indicated by top GO:BP term (orange boxes).

GO:0002181 cytoplasmic translation

GO:0043122 regulation of I-kappaB kinase/NF-kappaB signaling

GO:0007006 mitochondrial membrane organization

GO:0060070 canonical Wnt signaling pathway

GO:0051607 defense response to virus

GO:0007169 transmembrane receptor protein tyrosine kinase signalling pathway

GO:0048468 cell development

GO:0007565 female pregnancy

GO:0016055 Wnt signaling pathway

GO:0097237 cellular response to toxic substance

GO:0032479 regulation of type I interferon production

GO:0090522 vesicle tethering involved in exocytosis

GO:0075732 viral penetration into host nucleus
