# Supplementary material for: Predicting human and viral protein variants affecting COVID-19 susceptibility and repurposing therapeutics
Source: Sci Rep. 2024 Jun 20;14:14208. doi: 10.1038/s41598-024-61541-1 (PMC11190248; doi:10.1038/s41598-024-61541-1)
Supplement: Supplementary file 1 — Supplementary Information. [file 41598_2024_61541_MOESM1_ESM.zip › Supplementary files(allincludingrevised)_13May_2024/Supplementary file 8-Cavityplus-results.docx]

**Supplementary file 8:**

1. **CavityPlus results for IFIH1 (green):PLpro (orange) complex**


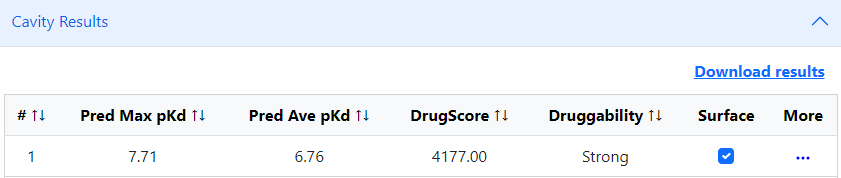

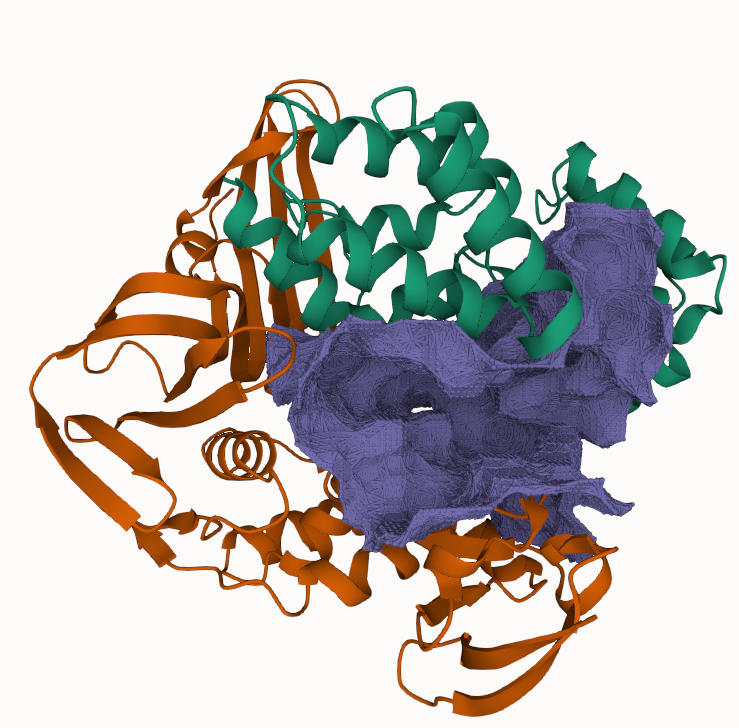


1. Cavityplus results for ARF6 (green):NSP15 (orange) complex


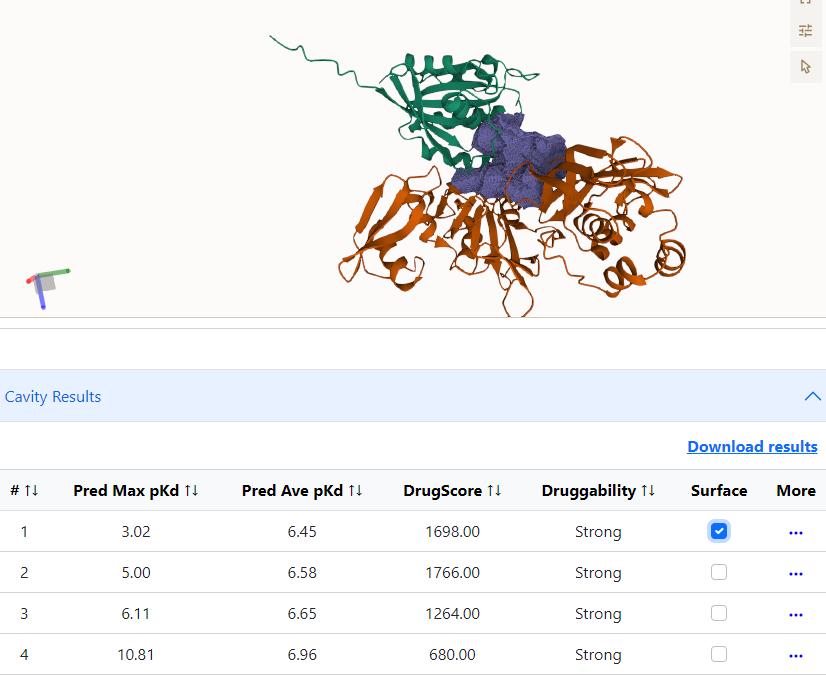


1. CavityPlus results for AXL:NTD complex


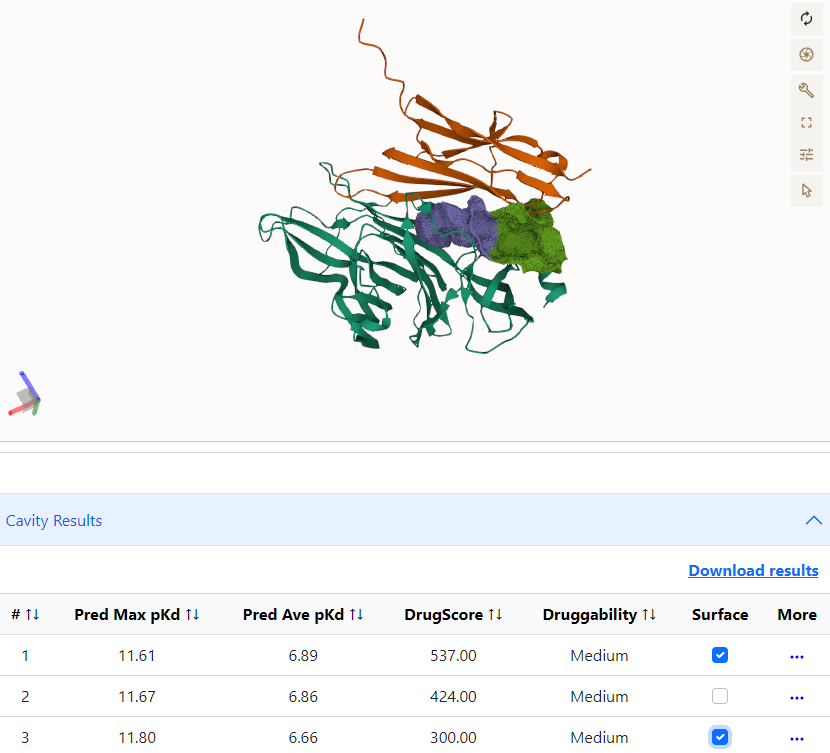


**Source of Cavity figures**: CavityPlus server output (<http://www.pkumdl.cn:8000/cavityplus/index.php#/> )

**CavityPlus reference:**

Wang, S., et al., *CavityPlus 2022 Update: An Integrated Platform for Comprehensive Protein Cavity Detection and Property Analyses with User-friendly Tools and Cavity Databases.* J Mol Biol, 2023. **435**(14): p. 168141.
