# Supplementary material for: Predicting human and viral protein variants affecting COVID-19 susceptibility and repurposing therapeutics
Source: Sci Rep. 2024 Jun 20;14:14208. doi: 10.1038/s41598-024-61541-1 (PMC11190248; doi:10.1038/s41598-024-61541-1)
Supplement: Supplementary file 1 — Supplementary Information. [file 41598_2024_61541_MOESM1_ESM.zip › Supplementary files(allincludingrevised)_13May_2024/Supplementary file 9-CASTp.docx]

**Supplementary file 9: CASTp prediction**

CASTp prediction for IFIH1:PLpro complex:


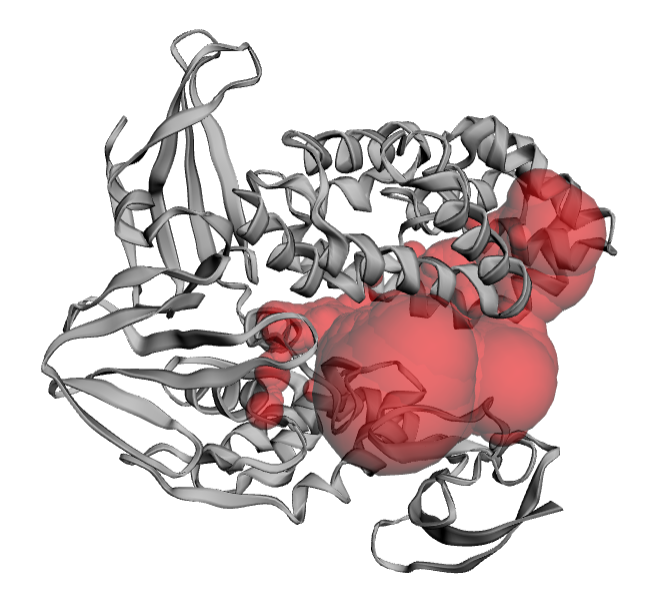


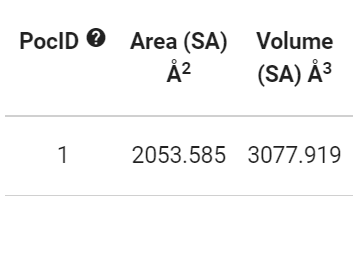


CASTp prediction for ARF6:NSP16 complex:


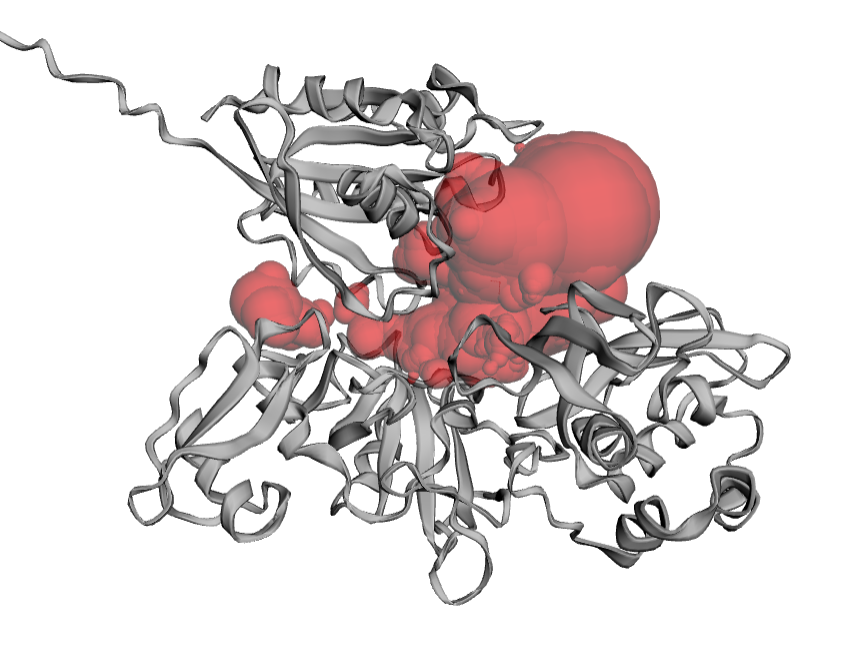


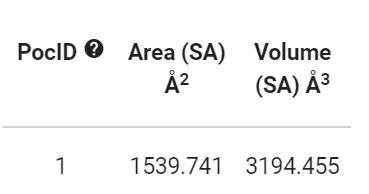


CASTp prediction for NTD:AXL interaction


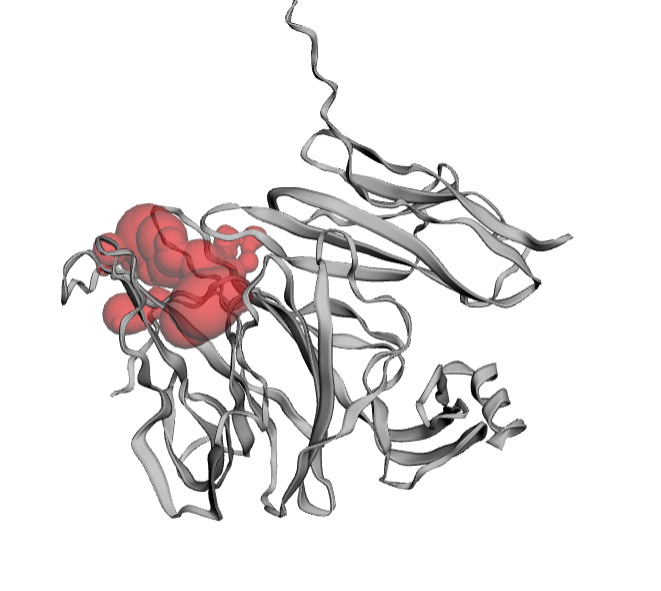


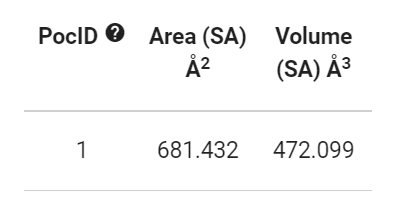


**Source of figures**: **CASTp** server output (<http://sts.bioe.uic.edu/castp/index.html?2pk9>)

**CASTp reference:**

Tian, Wei, Chang Chen, Xue Lei, Jieling Zhao, and Jie Liang. "CASTp 3.0: computed atlas of surface topography of proteins." *Nucleic acids research* 46, no. W1 (2018): W363-W367.
