# Supplementary material for: Predicting human and viral protein variants affecting COVID-19 susceptibility and repurposing therapeutics
Source: Sci Rep. 2024 Jun 20;14:14208. doi: 10.1038/s41598-024-61541-1 (PMC11190248; doi:10.1038/s41598-024-61541-1)
Supplement: Supplementary file 1 — Supplementary Information. [file 41598_2024_61541_MOESM1_ESM.zip › Supplementary files(allincludingrevised)_13May_2024/Supplementary Table 2-Dynamut2.docx]

**Supplementary Table 2**: Dynamut2 prediction on stability of human proteins.

| Name of the  human protein | SARS:CoV-2: Affinity-enhancing variants  in human proteins | **Dynamut2**  Gibbs free energy ΔΔG **(***kcal/mol*)  (< - 2 Kcal/mol: strongly destabilising [ Ref: Rodrigues et al., 2021] |
| --- | --- | --- |
| hTOMM70 | V514I | -0.23 |
|  | V556L | -0.46 |
|  | K576R | -0.55 |
|  | A591T | -1.87 |
|  | A483T | -1.99 |
| hISG15 | S21N | 0.02 (stabilising) |
|  | L121Q | -1.04 |
| hIFIH1 | S16L | 0.44 (stabilising) |
|  | Y13N | -1.84 |
| hIFIT2 | Y383F | -0.16 |
|  | K221E | -0.39 |
|  | A319S | -0.99 |
|  | L373F | -1.63 |
|  | A319T | -1.93 |
| hRPS3 | V164I | -0.04 |
|  | I99F | -0.84 |
| hNUP98 | T190S | *-0.47* |
| hARF6 | L166F | *-0.66* |
| hTRIM25 | A466T | *-1.56* |
| hTRIMM | I105F | ***-2.38*** |
| hAXL | V38M | *-0.52* |
| hACE2 | G326E* | -1.33 |
| hPALS1 | L321F | *-0.82* |
| hKremen1 | Y66H | -1.34 |
|  | V189I | -0.8 |

The cutoff of < -2 Kcal/mol (values shown in red), for strongly destabilising impact is referred from Rodrigues et al., 2021. Blue: Gibbs free energy ΔΔG > 0 Kcal/mol.

Dynamut2 reference: Rodrigues CHM, Pires DEV, Ascher DB. DynaMut2: Assessing changes in stability and flexibility upon single and multiple point missense mutations. Protein Sci. 2021 ;30(1):60-69. doi: 10.1002/pro.3942.
