# Supplementary material for: Predicting human and viral protein variants affecting COVID-19 susceptibility and repurposing therapeutics
Source: Sci Rep. 2024 Jun 20;14:14208. doi: 10.1038/s41598-024-61541-1 (PMC11190248; doi:10.1038/s41598-024-61541-1)
Supplement: Supplementary file 1 — Supplementary Information. [file 41598_2024_61541_MOESM1_ESM.zip › Supplementary files(allincludingrevised)_13May_2024/Supplementary Table1-metrics.docx]

**Supplementary Table 1: The 95% confidence interval for the mCSM-PPI2 predicted binding affinity values for all 26 affinity-enhancing variants from human proteins, reported in this study.**

| **Human protein** | **Coding variant** | **mCSM-PPI2 prediction(Kcal/mol)** | |
| --- | --- | --- | --- |
|  |  | **Upper CI value** | **Lower CI value** |
| **ACE2** | **G326E** | 0.993 | 0.882 |
| **TRIM25** | **A466T** | 0.61 | 0.523 |
| **IFIT2** | **K221E** | 0.848 | 0.659 |
|  | **L373F** | 0.804 | 0.616 |
|  | **Y383F** | 0.682 | 0.446 |
|  | **A319S** | 0.589 | 0.522 |
|  | **A319T** | 0.522 | 0.494 |
| **ARF6** | **L166F** | 0.663 | 0.612 |
| **NUP98** | **T190S** | 1.46 | 1.085 |
| **TRIMM** | **I105F** | 0.532 | 0.458 |
| **KREMEN1** | **Y66H** | 0.717 | 0.619 |
|  | **V189I** | 1.89 | 1.47 |
| **PALS1** | **L321F** | 0.8151 | 0.7168 |
| **AXL** | **V38M** | 0.535 | 0.432 |
| **IFIH1** | **Y13N** | 1.112 | 0.952 |
|  | **S16L** | 0.642 | 0.435 |
| **ISG15** | **LEU121GLN** | 0.659 | 0.516 |
|  | **SER21ASN** | 0.514 | 0.313 |
| **RPS3** | **VAL164ILE** | 0.699 | 0.406 |
|  | **ILE99PHE** | 0.580 | 0.526 |
| **TOM70** | **VAL514ILE** | 0.604 | 0.516 |
|  | **ALA483THR** | 0.629 | 0.500 |
|  | **LYS576ARG** | 0.866 | 0.732 |
|  | **VAL556LEU** | 1.037 | 0.945 |
|  | **ALA591THR** | 0.666 | 0.606 |

As can be seen from the table, for all of the 26 variants, we noted that either both CI values or upper CI value is above > 0.5 Kcal/mol.

Following steps were carried out to compute 95% confidence interval values:

We generated 10 different versions for each of the SARS-CoV-2:Human protein complex structure, using GalaxyrefineComplex software, which is specifically designed for structure refinement of protein-protein complexes. The refinement protocol involves both an initial local energy minimization and a 1.2-ps MD relaxation with a 4-fs time step. Thus, incorporation of MD relaxation step enables some physical space for atomic fluctuations, facilitating further conformational sampling. Particularly, relaxation of the input complex structure is driven by side-chain repacking of interfacial residues, which ensures overall conformational changes. Side-chain repacking and MD relaxation is repeated 22 times (13.2-ps) (please refer to Heo et al., 2016, for details).

For each variant, we calculated Mcsm-ppi2 scores using all the 10 versions of the models. In addition we also considered Mcsm-ppi2 score of the original reference complex. Thus, for the computation of 95% Confidence Interval values, sample size of 11, and alpha value of 0.05 was used. The average and standard deviation (SD) was obtained using Mcsm-ppi2 scores from 11 replicates, for each of the 26 variants. Finally, Confidence Interval was computed using formula:

Upper CI (95%): average + CONFIDENCE(0.05, SD ,11)

Upper CI (95%) : average - CONFIDENCE(0.05, SD ,11).

These values are provided for each of the 26 affinity-enhancing variants in the Supplementary Table 1, above.

**References:**

Heo, L., Lee, H. & Seok, C. GalaxyRefineComplex: Refinement of protein-protein complex model structures driven by interface repacking. Sci Rep 6, 32153 (2016). <https://doi.org/10.1038/srep32153>.

Carlos H M Rodrigues, Yoochan Myung, Douglas E V Pires, David B Ascher, mCSM-PPI2: predicting the effects of mutations on protein–protein interactions, Nucleic Acids Research, Volume 47, Issue W1, W338–W344, <https://doi.org/10.1093/nar/gkz383>
